# DUIE: Depth-Aware Underwater Image Enhancement via Spatial Prior Modeling

**DOI:** 10.3390/s26196138

**Published:** 2026-09-28

**Authors:** Haoyang Hong, Yuan Si, Yao Li, Chao Jiang, Chao Liang

**Affiliations:** 1 School of Computer Science, Wuhan University, Wuhan 430072, China; honghaoyang@whu.edu.cn (H.H.); ysi132@whu.edu.cn (Y.S.); cliang@whu.edu.cn (C.L.); 2 Naval University of Engineering, Wuhan 430033, China; 653462459@nue.edu.cn

**Keywords:** underwater image enhancement, depth-guided enhancement, depth prior modeling, pixel-level refinement, depth-weighted Laplacian pyramid fusion

## Abstract

**Highlights:**

**What are the main findings?**

- A depth prior modeling mechanism translates relative geometric depth into spatially continuous fusion weights for depth-adaptive underwater image enhancement.
- Two statistically enhanced candidates are refined and blended through depth-weighted Laplacian pyramids to preserve details and reduce abrupt transitions.

**What are the implications of the main findings?**

- Continuous depth-guided control alleviates the limitations of spatially uniform or discrete region-wise enhancement when degradation varies with scene depth.
- The single-RGB framework combines physics-inspired, statistical, and learned cues without requiring a full inversion of the underwater image formation model.

**Abstract:**

Underwater visual perception is degraded by wavelength-dependent absorption and scattering, the effects of which vary with the camera-to-scene distance. Many underwater image enhancement methods nevertheless apply image-level transformations or use region-wise statistics with discrete boundaries. Such control can make it difficult to preserve near-field appearance while improving far-field visibility, particularly when degradation changes gradually across the scene. We propose a Depth-Aware Underwater Image Enhancement (DUIE) framework that uses relative depth as a spatial prior for continuous enhancement control. DUIE first derives two complementary candidates through foreground- and background-specific statistical color compensation and refines both candidates with a lightweight Nonlinear Activation-Free (NAF)-based network. Its principal learned component jointly maps the input RGB image and an estimated relative depth map to a continuous pixel-wise fusion coefficient. A depth-weighted Laplacian pyramid then blends the refined candidates at multiple scales, replacing the binary mask fusion used by region-wise enhancement with smoother spatial control. The framework therefore combines a physics-inspired depth cue, interpretable statistical candidates, and learned local refinement without explicitly inverting a complete underwater image formation model. Experiments on three real underwater datasets show that DUIE obtains the best overall set of perceptual and edge-based scores among the compared methods, while qualitative results indicate balanced color correction, visibility, and structural detail.

## 1. Introduction

Marine and inland aquatic environments contain abundant resources, and underwater visual perception supports applications including Autonomous Underwater Vehicle (AUV) navigation, Simultaneous Localization and Mapping (SLAM), subsea inspection, and ecological observation [1,2,3,4]. Unlike imaging in air, underwater imaging is strongly affected by absorption and scattering in the participating medium, degrading both human observation and machine perception [5,6]. Underwater optical degradation is mainly governed by wavelength-dependent absorption and light scattering [7,8,9,10]. Red wavelengths are generally attenuated more rapidly than blue and green wavelengths, often producing blue–green color casts [11]. Forward scattering suppresses high-frequency structure, whereas backscattering introduces veiling light that lowers contrast and visibility. As shown in Figure 1, these effects can produce color distortion, haze, low contrast, and structural blur. Enhancement can improve visual interpretability, although improvements in image quality scores do not necessarily imply better downstream detection performance [2,12]. Underwater image enhancement (UIE) should therefore be evaluated as both an image restoration problem and a potential preprocessing step whose task-level benefit requires separate validation.

The image formation model in (4) and (5) indicates that transmission varies with the camera-to-scene distance and wavelength-dependent attenuation coefficient [7,8,10,13]. Physical model-based UIE methods estimate optical parameters and invert an assumed formation model [10,14,15]. Their interpretability is valuable, but errors in the assumed water properties, illumination, or parameter estimates can leave residual artifacts. Non-physical methods avoid full model inversion and are often more flexible, but many derive enhancement parameters from image-level or region-level statistics. When degradation changes gradually with distance, a single global operation or a small number of discrete regions can create a near–far conflict, wherein aggressive correction may alter near-field appearance, whereas conservative correction may leave distant regions hazy or color-biased. This observation motivates continuous spatial control but does not imply that an estimated monocular depth map is an exact measurement of optical attenuation.

To improve spatial adaptivity, UIE-SAM [16] uses a foundation segmentation model to separate foreground and background regions before region-specific color compensation. This reduces macroscopic color interference, but its fusion control is inherited from a discrete foreground–background mask. A binary partition is not designed to describe gradual changes in camera-to-scene distance, and its boundary may not coincide with the spatial progression of attenuation. Other studies have already introduced depth into learned UIE systems through relative depth attention, depth-conditioned generation, or near–far feature routing [17,18,19]. The remaining question is therefore not whether depth can be used, but how a relative depth estimate can regulate an interpretable enhancement process continuously while remaining tolerant of depth estimation errors.

To address this question, we propose a **D**epth-aware **U**nderwater **I**mage **E**nhancement (DUIE) framework, as illustrated in Figure 2. DUIE retains the interpretable foreground- and background-specific statistical candidates of UIE-SAM but replaces binary mask fusion with a learned continuous mixing coefficient. Specifically, a depth weight prediction module jointly processes the RGB image and a normalized relative depth estimate to produce a pixel-wise fusion map. In parallel, SAM-derived masks support the construction of two region-specific color-compensated candidates, which are passed through an NAF-based pixel refinement network. A depth-weighted Laplacian pyramid blends the refined candidates at multiple scales, and the inherited high-frequency fusion step supplies the final detail adjustment. In this organization, the statistical stage determines *what* complementary candidates are available, the refinement stage improves their local structure, and the RGB–depth module determines *where and how strongly* they are mixed. The contribution lies in this continuous multi-scale control of interpretable candidates, rather than in SAM, NAF blocks, or Laplacian pyramids individually.

In summary, the main contributions of this work are highlighted as follows:

- **Continuous RGB–Depth Fusion Control:** We jointly map the input RGB image and normalized relative depth to a continuous pixel-wise coefficient that controls the mixture of two complementary region statistics candidates. Unlike binary region masks, the coefficient supports gradual spatial variation while allowing image content to moderate an imperfect depth estimate.
- **Interpretable Coarse-to-Fine Organization:** We organize inherited region-adaptive color compensation and an NAF-based residual refiner into a coarse-to-fine pipeline. The two stages respectively address broad color imbalance and local structural residuals, preserving a clear functional separation between statistical correction and learned refinement.
- **Depth-Weighted Multi-Scale Synthesis:** We apply the predicted coefficient at every level of a Laplacian pyramid to synthesize the refined candidates. This formulation replaces hard mask compositing with scale-aware continuous blending and reduces abrupt transitions at region boundaries.
- **Multi-Aspect Evaluation:** Experiments on three real underwater datasets evaluate color contrast statistics, learned perceptual quality, and edge restoration. Among the compared methods, DUIE obtains the strongest overall metric profile, and ablations isolate the contributions of continuous depth weighting and pixel refinement.

## 2. Related Work

Existing underwater image enhancement methodologies can be broadly categorized into three primary paradigms: physical model-based methods, non-physical model-based methods, and deep learning-based methods [1,15].

### 2.1. Physical Model-Based Methods

Physical model-based methods describe underwater degradation through optical image formation models. They usually decompose the captured image into direct signal attenuation and backscattering components, and recover the latent scene radiance by estimating physical parameters such as transmission maps, background light, attenuation coefficients, or scene depth [5,6,7,8]. Owing to their explicit physical formulation, these methods offer strong interpretability and can preserve radiometric and structural cues, which is beneficial for downstream tasks involving measurement, geometric reasoning, or 3D reconstruction.

Early studies adapted atmospheric dehazing principles to underwater scenarios while accounting for wavelength-dependent light attenuation. Carlevaris-Bianco et al. [14] exploited inter-channel attenuation differences to estimate scene depth and reduce spatially varying haze. Chiang and Chen [10] proposed wavelength compensation and dehazing to compensate for attenuation discrepancies along the propagation path. Drews et al. [20] developed the underwater dark channel prior (UDCP), which estimates transmission by excluding the rapidly attenuated red channel, whereas Galdran et al. [21] proposed the Red Channel Prior (RCP) by explicitly exploiting red channel attenuation characteristics. Beyond direct adaptations of dehazing priors, subsequent studies introduced more underwater-specific optical modeling strategies. Song et al. [22] proposed the underwater light attenuation prior (ULAP) for scene depth estimation and transmission-related parameter inference. Berman et al. [23] modeled different water types using spectral profiles and formulated underwater color restoration through haze lines. Akkaynak and Treibitz [13] further proposed Sea-thru, a revised underwater image formation model that uses RGB-D information to estimate backscatter and range-dependent attenuation, providing an important reference for physically grounded underwater color recovery.

Recent methods have further explored hybrid frameworks that combine physical priors with learning-based models. Pan et al. [24] proposed LUIE, a learnable physical model-guided framework with bi-directional unsupervised domain adaptation. Wu et al. [25] introduced SLURPP, which combines pretrained latent diffusion priors with an explicit decomposition of attenuation and backscattering. Ma et al. [26] proposed ICAD-UIE, which is based on inter-channel attenuation differences, and Zhang et al. [27] used multi-angle polarization decomposition to separate polarized backscatter and target radiance.

Despite their interpretability, physical model-based methods often rely on simplified assumptions and accurate parameter estimation. Heterogeneous water types, non-uniform illumination, suspended particles, and multiple scattering can violate those assumptions and leave residual artifacts [5,6,27]. Explicitly estimating transmission, background light, or attenuation coefficients may also add computational cost. These limitations motivate methods that retain useful physical cues without requiring full model inversion.

### 2.2. Non-Physical Model-Based Methods

Non-physical model-based methods enhance underwater images without explicitly formulating or inverting the underwater imaging model. Instead, they rely on image statistics, color correction, histogram adjustment, Retinex decomposition, spatial filtering, and image fusion to improve color appearance and contrast [28,29,30]. Compared with physical model-based methods, these approaches are usually easier to implement and less dependent on precise optical parameter estimation.

Retinex-based methods enhance underwater images by decomposing illumination and reflectance components. Zhuang et al. [31] proposed a hyper-Laplacian reflectance prior-inspired Retinex model to improve structural details and color naturalness. Fusion-based methods construct multiple enhanced versions of the input and combine them with designed weight maps. Ancuti et al. [32] introduced a representative color balance and multi-scale fusion framework, which blends color-corrected and contrast-enhanced inputs to improve global visibility. Zhang et al. [33] proposed a minimum color loss principle with locally adaptive contrast enhancement to improve color preservation and local visibility. Kang et al. [34] developed a perception-aware decomposition and fusion framework that enhances underwater images in a perceptually motivated decomposition space.

Recent non-physical methods have further exploited underwater-specific statistical cues and foundation models. Wang et al. [35] explored underwater color disparities and used natural color consistency priors to guide adaptive color compensation. Wang et al. [16] introduced a SAM-empowered discriminative enhancement paradigm, where the Segment Anything Model is used to separate foreground and background regions so that region-specific adaptive color compensation can reduce foreground–background color cross-talk.

These non-physical methods are generally efficient and broadly applicable, since they do not require paired training data or explicit optical model inversion [15]. However, many of them derive enhancement parameters from image-level or region-level statistics. As a result, enhancement control is often spatially uniform or piece-wise discrete, limiting their ability to model the continuous variation of underwater degradation along scene depth. In particular, region-wise enhancement can reduce macroscopic color interference, but discrete foreground–background statistics may still be insufficient when degradation changes gradually across space. This motivates our use of a continuous depth prior to guide spatially adaptive enhancement and fusion.

### 2.3. Deep Learning-Based Methods

Deep learning-based methods use neural networks to learn nonlinear mappings from degraded images to enhanced outputs [15,36]. Their flexible representations can model complex image statistics, but their behavior depends on the supervision, training distribution, and architectural priors supplied to the network.

Early CNN approaches established representative baselines. UWCNN [37] used synthetic underwater data, whereas SGUIE-Net [38] incorporated semantic attention and multi-scale perception. FAnNet [39] proposed a fusion adversarial network and introduced the U45 test set, and PUGAN [40] incorporated image formation guidance into a dual-discriminator GAN. WaterNet [36] learned to combine conventionally enhanced inputs under human-selected UIEB references, while TUDA [41] addressed inter- and intra-domain gaps between synthetic and real underwater data.

Transformers and diffusion models have subsequently extended long-range and generative modeling. Boudiaf et al. [42] used a pretrained image processing Transformer; U-Shape Transformer [43] combined channel-wise multi-scale fusion with spatial global modeling; and HCLR-Net [44] introduced hybrid contrastive regularization. DiffWater [45] used degraded and color-compensated images as diffusion conditions.

Depth-guided enhancement is particularly relevant to DUIE. A depth-conditioned GAN concatenates an estimated depth map with RGB input inside a generator [18]. SARSDN learns relative depth attention together with semantic guidance in an end-to-end enhancement network [17]. UVZ predicts depth and uses it to route near- and far-field features through local and non-local perception branches [19]. These methods establish that depth guidance itself is not new. DUIE differs in terms of the role assigned to depth: the RGB–depth predictor does not directly reconstruct the output or route latent features; instead, it produces a continuous coefficient that mixes two explicit, region statistics-based candidates at every Laplacian scale. Relative to UIE-SAM [16], this replaces binary mask fusion while retaining the interpretability of the candidate construction.

Despite their representation capacity, learned methods remain sensitive to the quality and domain coverage of their training data [2,15]. Architectural or physical priors can improve interpretability but do not by themselves guarantee radiometric recovery or downstream task gains. Table 1 summarizes the input–output assumptions and trade-offs of the three broad families and positions DUIE as a single-RGB, multi-cue hybrid.

## 3. Preliminary

### 3.1. Underwater Imaging Model

McGlamery [8] and Jaffe [7] describe the irradiance reaching an underwater camera as the sum of direct transmission, forward scattering, and backscattering. For color channel c∈{r,g,b}, this decomposition is

(1) $$E_{\mathrm{total},c}\left(x\right)=E_{d,c}\left(x\right)+E_{f,c}\left(x\right)+E_{b,c}\left(x\right),$$

where *x* is a spatial location, $E_{d,c}$ is the attenuated signal reflected from the scene, $E_{f,c}$ accounts for signal scattered toward neighboring image locations, and $E_{b,c}$ is path radiance scattered into the camera. Following the commonly used single-image simplification, we omit $E_{f,c}$ in the subsequent model. This approximation does not imply that forward scattering is absent; it means that the present framework does not explicitly invert its spatial point-spread effect.

The direct attenuation component $E_{d}$ describes the light reflected by the object that successfully reaches the camera without being scattered away. It is expressed as

(2) $$E_{d,c}\left(x\right)=J_{c}\left(x\right)T_{c}\left(x\right),$$

where $J_{c}\left(x\right)$ is the latent scene radiance and $T_{c}\left(x\right)$ is the channel-dependent transmission.

The backscattering component $E_{b}$ represents the ambient light that is scattered directly into the camera lens by suspended particles in the water. This component creates a veil-like fog effect and is primarily responsible for the reduction in image contrast and color fidelity. It is defined as

(3) $$E_{b,c}\left(x\right)=B_{c}\left(1-T_{c}\left(x\right)\right),$$

where $B_{c}$ denotes the asymptotic background light component for channel *c* under the homogeneous-medium approximation.

By combining (2) and (3), the simplified yet widely adopted underwater optical model can be expressed as

(4) $$I_{c}\left(x\right)\approx E_{d,c}\left(x\right)+E_{b,c}\left(x\right)=J_{c}\left(x\right)T_{c}\left(x\right)+B_{c}\left(1-T_{c}\left(x\right)\right),$$

where $I_{c}\left(x\right)=E_{\mathrm{total},c}\left(x\right)$ is the recorded image intensity after the forward scattering term has been omitted. Transmission is commonly approximated by exponential decay [9,10]:

(5) $$T_{c}\left(x\right)=\exp\left[-\beta_{c}d\left(x\right)\right],$$

where $\beta_{c}$ is the channel-dependent attenuation coefficient and d(x) is the physical camera-to-scene distance. The monocular estimator used later predicts a normalized *relative* depth D(x) rather than d(x) or $T_{c}\left(x\right)$.

Equations (4) and (5) motivate distance-aware spatial control because transmission varies with d(x) [10,13,22]. DUIE uses this relationship as a design prior; it does not estimate $\beta_{c}$, $B_{c}$, or scene radiance by directly inverting the model.

### 3.2. NAF Module

We use the Nonlinear Activation-Free (NAF) block [46] as an existing lightweight feature transformation unit. This NAF element is not a contribution of DUIE; it is selected because its gated operations and residual paths provide a compact restoration backbone.

As illustrated in Figure 3, the first residual sub-block applies Layer Normalization, a 1×1 convolution, a 3×3 depth-wise convolution, SimpleGate, Simplified Channel Attention (SCA), and a final 1×1 convolution. SimpleGate, shown in Figure 3c, splits the channels into two equal parts and multiplies them element-wise. The result is scaled and added to the sub-block input.

SCA, shown in Figure 3b, aggregates spatial information by global average pooling and produces channel-wise modulation with a 1×1 convolution, avoiding the multi-layer attention branch used in squeeze-and-excitation networks [47]. A second residual sub-block then applies Layer Normalization, a 1×1 expansion, SimpleGate, and a 1×1 projection before another learned residual scaling. Including both residual sub-blocks is important because the NAF block is not only the SCA/SimpleGate sequence shown in the first branch.

The same block type is used to construct the depth weight predictor and the pixel-level refinement network, while the task-specific input–output mappings of those modules constitute the DUIE design.

## 4. Proposed Method

### 4.1. Architecture

Figure 2 shows the three functional stages of DUIE. First, a frozen underwater monocular depth estimator produces a normalized relative depth map, and a trainable RGB–depth predictor translates this geometric cue into a continuous fusion coefficient. Second, a frozen SAM provides a binary partition used by the inherited region-adaptive compensation procedure to construct two complementary candidates. Each candidate is processed by the NAF-based refinement mapping to recover local residual detail. Third, the continuous coefficient blends the candidates across Laplacian scales, which is followed by the inherited high-frequency fusion operation. The components are complementary rather than interchangeable: region statistics provide interpretable color correction alternatives, the refiner improves local structure within each alternative, and the RGB–depth map selects their spatial mixture without imposing the SAM boundary on the final fusion. A simpler direct use of relative depth would assume that normalized geometry is already an optical mixing coefficient; the learned mapping is introduced precisely because attenuation also depends on image content and unknown water properties.

The official pretrained weights of WsUID-Net and the SAM (ViT-H) are frozen during training and testing [48,49]; only the depth weight predictor and pixel-level refiner are optimized. Given the final output $I_{out}$ and the human-selected UIEB reference $I_{ref}$, the training objective is

(6) $$L_{\mathrm{MSE}}=\frac{1}{\left|\Omega \right|}\sum_{x\in \Omega }{∥I_{out}\left(x\right)-I_{ref}\left(x\right)∥}_{2}^{2},$$

where Ω is the image lattice and the norm is evaluated over RGB channels. The MSE supplies a stable paired pixel fidelity objective and jointly supervises the two trainable mappings through the reconstructed image. It does not, by itself, guarantee perceptual optimality or recovery of physically correct radiance; the physics-inspired behavior instead comes from constraining the learned output to a depth-controlled mixture of explicit candidates. Perceptual quality and structural change are assessed independently in Section 5. The UIEB reference is therefore treated as a perceptual training target rather than physical ground truth.

### 4.2. Depth Prior Modeling Mechanism

The depth prior modeling mechanism estimates normalized relative depth and translates it, together with RGB evidence, into a continuous fusion coefficient. The coefficient is learned for candidate mixing and should not be interpreted as a calibrated transmission or degradation probability map.

#### 4.2.1. Depth Estimation Module

The physical model in (5) links transmission to physical distance d(x), but a monocular image provides only relative geometric ordering. We therefore use relative depth as a structural cue rather than substituting it directly for d(x) or $T_{c}\left(x\right)$.

We adopt WsUID-Net [48], a weakly supervised model developed for single-image underwater relative depth estimation. Its training uses sparse depth order annotations together with semantic segmentation and edge-related auxiliary tasks to predict a dense relative map when metric depth supervision is scarce.

Formally, given a raw degraded underwater image $I\in R^{H\times W\times 3}$ with spatial dimensions H×W, the depth estimation process can be mathematically formulated as follows:

(7) $$D=F_{depth}\left(I;\Theta_{depth}\right),$$

where $F_{depth}\left(\cdot ;\Theta_{depth}\right)$ is the frozen estimator and $D\in {\left[0,1\right]}^{H\times W\times 1}$ is its normalized single-channel relative depth output.

The official WsUID-Net weights are used without fine-tuning. Thus, *D* supplies relative spatial ordering but may contain domain-dependent bias, boundary errors, or locally incorrect ordering. The following trainable module combines *D* with RGB content instead of treating it as a final fusion mask.

#### 4.2.2. Depth Weight Prediction Module

Normalized relative depth is not equivalent to optical attenuation because $\beta_{c}$, illumination, material reflectance, and estimation errors remain unknown. The depth weight prediction module therefore maps both image content and relative depth to a spatially continuous mixing coefficient:

(8) $$W=F_{weight}\left(I,D\right),$$

where $F_{weight}$ is trainable and $W\in {\left[0,1\right]}^{H\times W\times 1}$ controls the foreground candidate contribution. 1−W controls the background candidate in (30). This convention defines a fusion coefficient, not a physically calibrated degradation estimate.

Specifically, as illustrated in Figure 4, the raw degraded image *I* and its corresponding depth map *D* are first concatenated along the channel dimension to form a four-channel input feature *X*:

(9) X=[I,D].

A 3×3 convolutional layer $C_{in}$ projects this input into feature space:

(10) $$F_{0}=C_{in}\left(X\right).$$

The feature extractor contains $N_{w}$ NAF blocks [46]:

(11) $$F_{i+1}={\mathrm{NAF}}_{i}^{w}\left(F_{i}\right),\;i=0,\ldots ,N_{w}-1,$$

where the superscript distinguishes these blocks from those in the refinement network and $N_{w}$ is unrelated to the pyramid depth used later.

An output convolution maps $F_{N_{w}}$ to one channel:

(12) $$F_{out}=C_{out}\left(F_{N_{w}}\right).$$

A Sigmoid activation function is then applied to normalize the output into the range of [0,1]:

(13) $$W=\sigma (F_{out}),$$

where σ(·) denotes the Sigmoid function. The entire forward propagation process of this module can be summarized as follows:

(14) $$W=\sigma \left(C_{out}\left(H_{NAF}^{w}\left(C_{in}\left(\left[I,D\right]\right)\right)\right)\right),$$

where $H_{NAF}^{w}$ denotes the cascade of $N_{w}$ NAF blocks and both convolutions use 3×3 kernels.

RGB features give the predictor information with which to moderate unreliable or locally ambiguous depth cues. This design permits compensation for some depth errors, but does not guarantee that arbitrary depth failures are corrected. Section 5.8 evaluates bounded synthetic perturbations.

### 4.3. Coarse-to-Fine Regional Candidate Enhancement

This stage constructs the two interpretable candidates needed by the subsequent continuous fusion. It first applies the region-specific statistical compensation inherited from UIE-SAM and then uses a pixel-level residual network to refine the local structure in each candidate.

#### 4.3.1. Foreground–Background Extraction via the SAM

Following UIE-SAM [16], we use the frozen Segment Anything Model (SAM) [49] to obtain a category-agnostic partition for computing separate foreground and background color statistics. This partition is an inherited candidate construction device; it is not treated as a depth map or retained as the final fusion mask.

Specifically, the raw image *I* is processed by the SAM to generate a binary segmentation mask $M\in {\left\{0,1\right\}}^{H\times W}$. Based on this, the foreground mask $M^{F}$ and its complementary background mask $M^{B}$ are formalized as follows:

(15) $$M^{F}=M,\;M^{B}=1-M.$$

These complementary masks define the samples used to calculate the two sets of regional statistics. We do not further specify the internal candidate selection policy of the SAM because that detail was not available in the implementation record used for this manuscript.

#### 4.3.2. Foreground–Background Adaptive Color Compensation

We adopt the region-specific compensation procedure of Wang et al. [16]. Applied separately with $M^{F}$ and $M^{B}$, it produces two full-image candidates whose adjustment parameters are estimated from different regional samples. The four inherited steps are summarized below for reproducibility.

**(1) Reference-channel pre-compensation:** We first compute the regional mean intensity $\mu^{R}\left(I_{c}\right)$ for each color channel c∈{r,g,b} within region R∈{F,B}:

(16) $$\mu^{R}\left(I_{c}\right)=\frac{\sum_{i,j}I_{c}\left(i,j\right)M^{R}\left(i,j\right)}{\sum_{i,j}M^{R}\left(i,j\right)}.$$

The pixel-wise maximum channel is used as a reference:

(17) $$I^{max}=\max\left[I_{r},I_{g},I_{b}\right],$$

where max[·] denotes the pixel-wise maximum across the three channels. The pre-compensated image $R^{I}$ for region *R* is formulated as follows:

(18) $$\begin{array}{cc} R_{r}^{I} & =I_{r}\\ R_{b}^{I} & =I_{b}+I\left[\mu^{R}\left(I_{b}\right)>\mu^{R}\left(I_{g}\right)\right]\left(\mu^{R}\left(I^{max}\right)-\mu^{R}\left(I_{b}\right)\right)I^{max}\\ R_{g}^{I} & =I_{g}+I\left[\mu^{R}\left(I_{b}\right)\leq \mu^{R}\left(I_{g}\right)\right]\left(\mu^{R}\left(I^{max}\right)-\mu^{R}\left(I_{g}\right)\right)I^{max}, \end{array}$$

where I[·] is the indicator function.

**(2) Green–blue adaptive compensation:** The procedure uses target inter-channel histogram correlations as natural-image statistical priors [16,50]. Let $S_{c_{1}c_{2}}^{R}$ be the Pearson correlation between the regional intensity histograms of channels $c_{1}$ and $c_{2}$, and let $S_{c_{1}c_{2}}^{N}$ be the target prior. The green and blue channels are updated toward $S_{gb}^{N}$:

(19) $$\begin{array}{cc} R_{g}^{II}= & R_{g}^{I}+I\left[\mu^{R}\left(R_{b}^{I}\right)>\mu^{R}\left(R_{g}^{I}\right)\right]\left(\alpha +S_{gb}^{N}-S_{gb}^{R}\right)\\ & \times \left(\mu^{R}\left(R_{b}^{I}\right)-\mu^{R}\left(R_{g}^{I}\right)\right)R_{b}^{I}\\ R_{b}^{II}= & R_{b}^{I}+I\left[\mu^{R}\left(R_{b}^{I}\right)\leq \mu^{R}\left(R_{g}^{I}\right)\right]\left(\alpha +S_{gb}^{N}-S_{gb}^{R}\right)\\ & \times \left(\mu^{R}\left(R_{g}^{I}\right)-\mu^{R}\left(R_{b}^{I}\right)\right)R_{g}^{I}, \end{array}$$

where α is a parameter controlling the compensation intensity.

**(3) Red–green adaptive compensation:** The compensated green channel is then used to update the more strongly attenuated red channel toward $S_{rg}^{N}$:

(20) $$R_{r}^{II}=R_{r}^{I}+\left(\alpha +S_{rg}^{N}-S_{rg}^{R}\right)\left(\mu^{R}\left(R_{g}^{II}\right)-\mu^{R}\left(R_{r}^{I}\right)\right)R_{g}^{II}.$$

**(4) Contrast stretching:** Finally, to expand the dynamic range and improve the local contrast of the compensated image, we apply a linear contrast stretching operation:

(21) $$R_{c}^{III}=\frac{\mathrm{clip}\left[R_{c}^{II},q_{\gamma }\left(R_{c}^{II}\right),q_{1-\gamma }\left(R_{c}^{II}\right)\right]-q_{\gamma }\left(R_{c}^{II}\right)}{q_{1-\gamma }\left(R_{c}^{II}\right)-q_{\gamma }\left(R_{c}^{II}\right)},$$

where $q_{\gamma }\left(\cdot \right)$ denotes the quantile function at order γ, and clip[·] bounds the pixel intensities to the desired percentile range.

The iterations stop when the target is reached or when the correlation improvement is below $10^{-4}$, preventing non-convergent updates. The outputs are the foreground statistics candidate $F^{III}$ and background statistics candidate $B^{III}$. Algorithm 1 summarizes this inherited procedure.
**Algorithm 1** Foreground–Background Adaptive Color Compensation**Input:** Raw underwater image *I*, Semantic masks $M^{F}$ and $M^{B}$, Parameters α,γ, Natural color histogram similarities $S_{gb}^{N},S_{rg}^{N}$.**Output:** Coarse foreground enhanced image $F^{III}$ and background enhanced image $B^{III}$.  1:Compute reference channel $I^{max}=\max\left[I_{r},I_{g},I_{b}\right]$.  2:**for each** region R∈{F,B} **do**  3:   Calculate regional means $\mu^{R}\left(I_{r}\right),\mu^{R}\left(I_{g}\right),\mu^{R}\left(I_{b}\right)$ using (16).  4:   Obtain pre-compensated channels $R_{r}^{I},R_{g}^{I},R_{b}^{I}$ using (18).  5:   Calculate initial Pearson correlation $S_{gb}^{R}$ between $R_{g}^{I}$ and $R_{b}^{I}$.  6:   **while** $S_{gb}^{R}<S_{gb}^{N}$ and the correlation increment is at least $10^{-4}$ **do**  7:     Update $R_{g}^{II}$ and $R_{b}^{II}$ using (19).  8:     Assign $R_{g}^{I}\leftarrow R_{g}^{II}$, $R_{b}^{I}\leftarrow R_{b}^{II}$ and recompute $S_{gb}^{R}$.  9:   **end while**10:   Calculate initial Pearson correlation $S_{rg}^{R}$ between $R_{r}^{I}$ and $R_{g}^{II}$.11:   **while** $S_{rg}^{R}<S_{rg}^{N}$ and the correlation increment is at least $10^{-4}$ **do**12:     Update $R_{r}^{II}$ using (20).13:     Assign $R_{r}^{I}\leftarrow R_{r}^{II}$ and recompute $S_{rg}^{R}$.14:   **end while**15:   Obtain full compensated image $R^{II}=\left\{R_{r}^{II},R_{g}^{II},R_{b}^{II}\right\}$.16:   **for each** channel c∈{r,g,b} **do**17:     Apply contrast stretching to compute $R_{c}^{III}$ using (21).18:   **end for**19:**end for**20:**return** $F^{III}$, $B^{III}$

#### 4.3.3. Pixel-Level Refinement Module

Region statistics compensation adjusts channel distributions and contrast but does not explicitly model local spatial residuals. The pixel-level refinement module therefore processes each compensated candidate before fusion, allowing learned corrections of textures, boundaries, and local artifacts that are not represented by the histogram statistics.

As illustrated in Figure 5, the module is a U-Net-style encoder–decoder [51] built from NAF blocks [46]. Its input is $R^{III}$, where R∈{F,B} denotes either candidate. The notation below applies the same architectural mapping to both candidates without making an unsupported claim about parameter sharing between branches.

The refinement process can be formulated as a global residual learning framework [52,53]:

(22) $$R^{IV}=R^{III}+F_{\mathrm{refine}}\left(R^{III}\right),$$

where $F_{\mathrm{refine}}$ is the encoder–decoder residual mapping and $R^{IV}$ is the refined candidate.

An initial 3×3 convolution maps the RGB candidate to the full-resolution feature $E_{0}$:

(23) $$E_{0}=C_{init}\left(R^{III}\right).$$

The encoder has four stages. At stage *i*, a stride-2 convolution reduces the spatial resolution and an NAF cascade $H_{i}^{e}$ transforms the features:

(24) $$E_{i}=H_{i}^{e}\left(C_{↓,i}\left(E_{i-1}\right)\right),\;i=1,2,3,4.$$

The four encoder cascades contain [1,1,1,28] NAF blocks, respectively, and $E_{4}$ is the bottleneck representation.

The decoder uses four PixelShuffle upsampling operations [54], avoiding transposed convolution checkerboard patterns [55]. Let $D_{4}=E_{4}$. Each decoder stage adds the corresponding encoder feature and applies one NAF block $H_{i}^{d}$:

(25) $$D_{i}=H_{i}^{d}\left(P_{i}\left(D_{i+1}\right)+E_{i}\right),\;i=3,2,1,0,$$

where the decoder block sequence is [1,1,1,1] from coarse to fine.

Ultimately, a final 3×3 convolution $C_{final}$ projects the deep features back to the three-channel image space, which is then added to the original input $R^{III}$ via the global skip connection, as defined in (22). The final fine-grained output can be rewritten as

(26) $$R^{IV}=C_{final}\left(D_{0}\right)+R^{III}.$$

The global skip connection biases the network toward residual correction rather than reconstruction from scratch [53]. The residual is not constrained to be strictly high-frequency; its contribution to perceptual and edge-related scores is evaluated by ablation.

### 4.4. Depth Prior-Guided Fusion Strategy

The fusion stage applies the learned coefficient to the two refined candidates at multiple scales and then uses an inherited high-frequency operation for final detail adjustment.

#### 4.4.1. Depth-Weighted Laplacian Fusion

Because the candidates are generated from different regional statistics, binary single-scale compositing can expose seams or halos, and excessive feathering can blur boundaries. Laplacian pyramid blending [32,56,57] decomposes the candidates into frequency bands so that low-frequency appearance and higher-frequency structure can be mixed at their corresponding scales.

UIE-SAM [16] uses its discrete segmentation mask for fusion. DUIE instead uses the continuous learned coefficient *W*. Because *W* is derived from estimated relative depth and RGB content, this change provides depth-informed spatial control but should not be interpreted as recovery of metric depth or physically exact transmission.

The overall procedure of the proposed depth-weighted Laplacian pyramid fusion is illustrated in Figure 6. The specific process is detailed below.

(1) *Pyramid extraction:* Let K∈{F,B,W} index the refined candidates $X^{F}=F^{IV}$ and $X^{B}=B^{IV}$ and the coefficient $X^{W}=W$. Their Gaussian pyramids are

(27) $$GP_{0}^{K}=X^{K},\;GP_{n}^{K}=\mathrm{Reduce}\left(GP_{n-1}^{K}\right),\;n=1,\ldots ,L,$$

where Reduce denotes low-pass filtering followed by downsampling and *L* is the maximum pyramid level.

For R∈{F,B}, the expansion of level n+1 to the spatial size of level *n* is defined as

(28) $${\tilde{GP}}_{n+1\to n}^{R}={\mathrm{Expand}}_{n+1\to n}\left(GP_{n+1}^{R}\right),\;n=0,\ldots ,L-1.$$

We then construct the Laplacian pyramids $LP_{n}^{R}$, which capture the differences between adjacent Gaussian levels:

(29) $$LP_{n}^{R}=GP_{n}^{R}-{\tilde{GP}}_{n+1\to n}^{R},\;n=0,\ldots ,L-1;\;LP_{L}^{R}=GP_{L}^{R}.$$

The band-pass layers represent scale-dependent detail, while the top residual represents the coarsest appearance component [56,57].

(2) *Layer-wise fusion:* At each scale,

(30) $$LP_{n}=GP_{n}^{W}⊙LP_{n}^{F}+\left(1-GP_{n}^{W}\right)⊙LP_{n}^{B},$$

where ⊙ is element-wise multiplication. Thus, the stated convention is explicit: $GP_{n}^{W}$ weights the foreground statistics candidate and $1-GP_{n}^{W}$ weights the background statistics candidate.

(3) *Pyramid reconstruction:* Let ${\hat{G}}_{n}$ denote a reconstructed level, distinct from the input Gaussian pyramids. Reconstruction proceeds from coarse to fine:

(31) $${\hat{G}}_{L}=LP_{L},\;{\hat{G}}_{n}=LP_{n}+{\mathrm{Expand}}_{n+1\to n}\left({\hat{G}}_{n+1}\right),\;n=L-1,\ldots ,0.$$

The fused image is $I^{V}={\hat{G}}_{0}$, equivalently,

(32) $$\begin{array}{cc} I^{V} & =LP_{0}+\mathrm{Expand}(LP_{1}+\mathrm{Expand}(LP_{2}\\ & \;+\cdots +\mathrm{Expand}(LP_{L}))). \end{array}$$

The complete process is formalized in Algorithm 2.
**Algorithm 2** Depth-Weighted Laplacian Pyramid Fusion**Input:** Refined foreground image $F^{IV}$, refined background image $B^{IV}$, continuous fusion coefficient *W*, maximum pyramid level *L*.**Output:** Fused image $I_{fused}$.  1:Initialize base levels: $GP_{0}^{F}=F^{IV}$, $GP_{0}^{B}=B^{IV}$, $GP_{0}^{W}=W$.  2:**for** n=1 to *L* **do**  3:   Compute Gaussian layers $GP_{n}^{R}=\mathrm{Reduce}\left[GP_{n-1}^{R}\right]$ for R∈{F,B,W}.  4:**end for**  5:**for** n=0 to L−1 **do**  6:   Compute expanded layers ${\tilde{GP}}_{n+1}^{R}=\mathrm{Expand}\left[GP_{n+1}^{R}\right]$ for R∈{F,B}.  7:   Construct Laplacian layers $LP_{n}^{R}=GP_{n}^{R}-{\tilde{GP}}_{n+1}^{R}$ for R∈{F,B}.  8:**end for**  9:Assign top layers: $LP_{L}^{F}=GP_{L}^{F}$, $LP_{L}^{B}=GP_{L}^{B}$.10:**for** n=0 to *L* **do**11:   Fuse Laplacian layers: $LP_{n}=GP_{n}^{W}⊙LP_{n}^{F}+\left(1-GP_{n}^{W}\right)⊙LP_{n}^{B}$.12:**end for**13:Initialize reconstruction: ${\hat{G}}_{L}=LP_{L}$.14:**for** n=L−1 down to 0 **do**15:   Reconstruct ${\hat{G}}_{n}=LP_{n}+{\mathrm{Expand}}_{n+1\to n}\left({\hat{G}}_{n+1}\right)$.16:**end for**17:**return** Fused image $I_{fused}={\hat{G}}_{0}$.

#### 4.4.2. High-Frequency Edge Fusion

We retain the high-frequency edge fusion procedure of Wang et al. [16,50]. This inherited postprocessing step injects a high-pass residual from the input and applies bounded edge-dependent contrast modulation.

The high-pass residual of the input is added to $I^{V}$:

(33) $${\tilde{I}}_{c}^{V}=I_{c}^{V}+\left(I_{c}-\mathrm{Gauss}\left[I_{c},\sigma \right]\right),\;c\in \left\{r,g,b\right\}$$

where Gauss[·,σ] is Gaussian filtering with standard deviation σ.

The edge control map is clipped to the valid intensity interval:

(34) $$E_{c}={\mathrm{clip}}_{\left[0,1\right]}\left({\tilde{I}}_{c}^{V}-\mathrm{Gauss}\left[{\tilde{I}}_{c}^{V},\sigma \right]+\theta \right),\;c\in \left\{r,g,b\right\}.$$

The parameter θ centers the modulation and is also the threshold in the following piece-wise mapping:

(35) $$I_{c}^{\mathrm{VI}}=\left\{\begin{array}{cc} \frac{{\tilde{I}}_{c}^{V}E_{c}}{\theta }, & \mathrm{if}\;E_{c}<\theta \\ 1-\frac{\left(1-{\tilde{I}}_{c}^{V}\right)\left(1-E_{c}\right)}{\theta }, & \mathrm{otherwise} \end{array}\right.\;c\in \left\{r,g,b\right\}$$

where θ serves as both a compensation parameter and a threshold for the blending function. This high-frequency edge enhancement result $I^{\mathrm{VI}}$ serves as the ultimate output $I_{out}$ of the entire enhancement framework.

## 5. Experiments

This section evaluates DUIE against twelve representative learning-based and prior-based methods. We first describe the implementation, datasets, and complementary evaluation criteria, and then report quantitative, qualitative, ablation, and depth perturbation results on U45, SUIM, and Sea-thru.

### 5.1. Experimental Setup

The framework is implemented in PyTorch 2.14.0 and trained on a single NVIDIA GeForce RTX 3090 GPU with 24 GB of memory. The SAM ViT-H model and WsUID-Net depth estimator are frozen; only the depth weight prediction and pixel-level refinement networks are optimized. For each full-resolution training image, we first obtain the relative depth map, segmentation mask, and two coarse enhanced candidates. The RGB image, depth map, candidates, and reference are then cropped synchronously to 256×256 pixels. This ordering preserves pixel-wise alignment and allows the frozen depth estimator to observe the complete scene before the trainable networks are optimized on local patches.

We use Adam [58] with a batch size of 1, $\beta_{1}=0.9$, $\beta_{2}=0.999$, and an initial learning rate of $5\times 10^{-4}$. The learning rate is halved every 100,000 iterations, the gradient norm is clipped to 1.0, and training runs for 500,000 iterations with random seed 1234 and four data-loading workers. Checkpoints are saved every 5000 iterations. The reported model is the saved checkpoint with the lowest MSE on a held-out validation subset of the UIEB reference set; we do not specify a split size because that quantity was not retained in the current experiment record. At inference, an image is processed at its original resolution.

For foreground–background adaptive color compensation, we follow the empirical configuration of Wang et al. [16]: α=1, γ=0.02, $S_{gb}^{N}=0.92$, and $S_{rg}^{N}=0.99$. The maximum Laplacian pyramid level is L=5. In the inherited high-frequency fusion step, the Gaussian standard deviation is σ=5, and the compensation threshold is θ=0.5. These values are held fixed in all reported evaluations.

### 5.2. Comparative Methods

The comparison set comprises twelve representative underwater image enhancement methods spanning learned and prior-based paradigms. The learning-based methods are U-shape [43], PUGAN [40], RLUIE [59], SMDR-IS [60], HCLR [44], FUSION [61], FAnNet [39], UIE-SAM [16], and Semi-UIR [62]; the prior-based methods are UDCP [20], RCP [21], and ULAP [22].

UIE-SAM [16] is the closest baseline because it also uses SAM-derived regional statistics, but it fuses the regional results using a binary semantic partition. The remaining learned methods provide different comparison points: U-shape employs a Transformer architecture [43]; PUGAN uses adversarial learning with physical guidance [40]; RLUIE formulates enhancement as reinforcement learning [59]; SMDR-IS and HCLR use multi-scale detail refinement and contrastive regularization, respectively [44,60]; FUSION performs frequency-guided spatial reconstruction [61]; FAnNet learns adversarial fusion among conventionally enhanced inputs [39]; and Semi-UIR uses a mean teacher framework with a reliable bank and contrastive regularization [62]. UDCP, RCP, and ULAP estimate degradation or scene depth-related quantities from hand-crafted underwater priors rather than learned mappings [20,21,22]. Together, these baselines support comparison across learned and prior-based design families without implying exhaustive coverage of all UIE methods.

### 5.3. Datasets

To comprehensively evaluate the generalization ability and robustness of our proposed framework, we utilize the Underwater Image Enhancement Benchmark (UIEB) for training [36] and three widely adopted public datasets for testing: the Underwater Test Dataset (U45) [39], the Segmentation of Underwater IMagery (SUIM) [63], and the Sea-thru dataset [13].

The UIEB reference subset contains 890 real underwater images paired with enhanced reference images selected through human evaluation [36]. We partition this paired subset into training and held-out validation data and use the latter only for checkpoint selection. These references provide perceptual paired supervision, but they are not measurements of the physically true scene radiance.

For the testing phase, we conduct extensive evaluations on the U45, SUIM, and Sea-thru datasets to assess the model’s performance across different degradation scenarios.

**(1)**  **U45 Dataset:** The U45 dataset [39] contains 45 representative real underwater images. It is categorized into three distinct subsets: green, blue, and haze-like. These subsets correspond to images suffering from green color deviations, blue color deviations, and severe haze blurring effects, respectively. This dataset is primarily utilized to evaluate the performance of different algorithms in terms of accurate color cast correction and robust dehazing.
**(2)**  **SUIM Dataset:** The SUIM dataset [63] is a large-scale collection consisting of 1635 annotated underwater images. It encompasses a wide variety of underwater objects and salient targets, including marine organisms, human divers, and underwater robots. We leverage this dataset to assess the effectiveness of the enhancement methods in preserving local details and improving the visual discernibility of underwater targets.
**(3)**  **Sea-thru Dataset:** The Sea-thru dataset [13] consists of real-world underwater images accompanied by their corresponding depth maps, primarily captured during dives in shallow coastal environments. Characterized by severe color distortion and sharp contrast degradation induced by complex water scattering and absorption, this dataset serves as a rigorous benchmark for evaluation of the algorithms’ restoration capabilities and robustness under highly challenging aquatic conditions.

### 5.4. Evaluation Metrics

We report five complementary no-reference or blind metric families, yielding six scalar indicators: UCIQE [64], UIQM [65], URanker [66], MUSIQ [67], and the two BCRA descriptors [68]. UCIQE is computed in CIELab space as

(36) $$\mathrm{UCIQE}=0.4680\sigma_{c}+0.2745{\mathrm{con}}_{l}+0.2576\mu_{s},$$

where $\sigma_{c}$ is the standard deviation of chroma, ${\mathrm{con}}_{l}$ is the luminance contrast, and $\mu_{s}$ is the mean saturation. A high UCIQE therefore indicates a strong combination of chromatic dispersion, saturation, and luminance contrast; it does not by itself establish color naturalness or restoration fidelity.

UIQM combines underwater colorfulness (UICM), sharpness (UISM), and contrast (UIConM) according to the following equation [65]:

(37) UIQM=0.0282UICM+0.2953UISM+3.5753UIConM.

Larger UIQM values indicate a stronger aggregate of these three attributes, but the linear combination cannot determine whether colors are physically correct.

URanker is a learned underwater-specific ranking model, whereas MUSIQ is a multi-scale Transformer-based general image quality model [66,67]. We use their fixed pretrained scorers without task-specific fine-tuning. Their outputs have no closed-form image statistics interpretation, and disagreement between them can arise from their different training data and quality preferences.

For an original image $I_{o}$ and its restored image $I_{r}$, let $n_{o}$ and $n_{r}$ denote their visible-edge counts and $P_{r}$ the restored image edge set. BCRA reports

(38) $$\mathrm{BC}\left(e\right)=\frac{n_{r}-n_{o}}{n_{o}},\;\mathrm{BC}\left(r\right)=\exp\left(\frac{1}{n_{r}}\sum_{p\in P_{r}}\log r\left(p\right)\right),$$

where $r\left(p\right)=f^{-1}\left(\Delta I_{r}\left(p\right)\right)/f^{-1}\left(\Delta I_{o}\left(p\right)\right)$ is the gradient ratio at *p*, ΔI denotes the local edge gradient, and *f* is the camera response function. The implementation follows the visible-edge detector in the original method [68]. BC(e) measures the relative change in visible-edge count, so it may be negative, while BC(r) is the geometric mean of gradient ratios. Larger values indicate stronger edge visibility or contrast amplification, not necessarily fewer artifacts or greater physical fidelity. We therefore interpret the reported indicators jointly rather than treating any single metric as conclusive.

### 5.5. Quantitative Comparisons

Table 2, Table 3 and Table 4 report the results of recent learning-based approaches and representative prior-based methods. Six no-reference or blind indicators are used: UCIQE and UIQM describe complementary color contrast aggregates, URanker and MUSIQ provide learned quality assessments, and BC(e) and BC(r) characterize visible-edge changes. An upward arrow indicates that a larger value is preferred by the corresponding metric; nevertheless, the distinct scope of each metric described in Section 5.4 must be retained when interpreting the rankings.

#### 5.5.1. Performance on the U45 Dataset

U45 contains blue-, green-, and haze-dominated images. DUIE ranks first in UIQM (3.331), MUSIQ (53.547), BC(e) (1.471), and BC(r) (4.725) and second in UCIQE (0.462) and URanker (2.611). UIE-SAM attains the highest UCIQE (0.463) and URanker (2.797), exceeding DUIE by 0.001 and 0.186, respectively. Conversely, DUIE exceeds UIE-SAM by 0.651 in UIQM, 1.169 in MUSIQ, 0.088 in BC(e), and 0.054 in BC(r). The reversal is informative: UIE-SAM is favored by the underwater-specific ranking model and marginally by the UCIQE color contrast combination, whereas DUIE receives stronger scores from the broader UIQM aggregate, the multi-scale learned quality model, and both edge visibility descriptors. The result therefore supports a balanced advantage across complementary criteria rather than uniform dominance.

The comparison also reveals clear differences across the learned and prior-based families. Among the three hand-crafted priors, ULAP produces the highest UCIQE (0.432), MUSIQ (47.068), and BC(e) (0.889), while UDCP gives the highest UIQM (2.313), URanker (1.001), and BC(r) (2.266); all remain below the strongest learned results for these respective criteria. FAnNet obtains a relatively strong MUSIQ (49.741) but a lower UCIQE (0.301) and URanker (0.487), illustrating that multi-scale perceptual structure need not coincide with underwater-specific color and ranking preferences. Semi-UIR is competitive in UIQM (3.251) and URanker (2.165), but DUIE retains margins of 0.080 and 0.446 and also produces higher MUSIQ and BCRA values. Taken together, the U45 results favor DUIE under several complementary criteria but do not establish physical color recovery or allow the system-level gains to be assigned to an individual module. Component-level evidence is examined in Section 5.7.

#### 5.5.2. Performance on the SUIM Dataset

SUIM contains diverse semantic objects and backgrounds. DUIE ranks first in UIQM (2.782), MUSIQ (61.963), BC(e) (1.563), and BC(r) (3.918), and second in URanker (2.065), below UIE-SAM (2.182). Its UIQM exceeds the second-ranked RLUIE result by 0.098, Semi-UIR by 0.163, FAnNet by 0.404, and the strongest prior-based value, obtained by UDCP, by 0.626. For MUSIQ, FAnNet is the closest competitor at 61.231, leaving a margin of 0.732. These results indicate a favorable balance between underwater colorfulness, sharpness, contrast, and multi-scale perceptual structure across the heterogeneous SUIM scenes.

The ordering remains metric-dependent. RLUIE has the highest UCIQE (0.532), followed by UIE-SAM (0.482), while DUIE ranks third with 0.477. Conversely, UIE-SAM is preferred by the underwater-specific URanker, whereas DUIE obtains higher UIQM, MUSIQ, and BCRA values. This reversal reflects the different attributes emphasized by the metrics rather than an internal inconsistency: UCIQE aggregates chroma dispersion, saturation, and luminance contrast; URanker follows an underwater-specific learned preference; and MUSIQ evaluates multi-scale perceptual structure. DUIE’s BCRA margins over UIE-SAM are +0.259 for BC(e) and +0.183 for BC(r), indicating greater edge count and gradient amplification for that assessment, although BCRA cannot determine whether every additional or strengthened edge is artifact-free.

The prior-based results provide a further reference point. ULAP gives the strongest prior-based UCIQE (0.474) and BC(e) (0.651), whereas UDCP gives the strongest prior-based UIQM (2.156), URanker (1.187), MUSIQ (57.333), and BC(r) (1.869). DUIE exceeds the prior-based maximum on all six metrics; its UCIQE margin is only 0.003 over ULAP, however, and it remains below the two leading learned methods on that criterion. Overall, the SUIM evidence supports a broadly balanced enhancement result rather than dominance under every individual criterion.

#### 5.5.3. Performance on the Sea-Thru Dataset

Sea-thru contains images affected by pronounced range-dependent attenuation and heterogeneous water conditions. Table 4 reports the recomputed results for all methods on the same 1216-image set. DUIE achieves the best value for four of the six indicators, the second-best MUSIQ value, and the third-best UCIQE value. This profile permits a more informative assessment than a claim based on any single score: the four leading values reflect complementary underwater-specific, learned, and edge-based criteria, while the remaining ordering still reveals metric-dependent preferences.

DUIE obtains the highest UIQM (3.209) and URanker (2.949). Relative to the respective second-ranked results, it improves UIQM compared to U-shape by 0.065 and URanker compared to UIE-SAM by 0.085. The former summarizes colorfulness, sharpness, and contrast, whereas the latter represents an underwater-specific learned preference. Agreement between these two differently constructed criteria provides complementary evidence that the result is not favored only by a single hand-crafted statistic. DUIE also achieves the largest BC(e) (0.141) and BC(r) (18.647), exceeding UIE-SAM by 0.016 and 1.061, respectively. Under the BCRA definitions, these margins indicate greater increases in the visible-edge count and edge gradient ratio relative to the inputs; they should not, however, be interpreted as proof that every strengthened edge is artifact-free.

The remaining two indicators expose a useful trade-off. DUIE ranks second in MUSIQ at 44.019, below HCLR (47.259) but above RCP (42.002). Thus, the general learned image quality model also rates DUIE favorably, although its preference differs from the underwater-specific URanker. For UCIQE, UDCP (0.524) and UIE-SAM (0.521) rank ahead of DUIE (0.510), with small margins of 0.013 and 0.011. Because UCIQE is driven by chroma dispersion, saturation, and luminance contrast, this ordering does not contradict the results for UIQM, URanker, MUSIQ, or BCRA; rather, it shows that the methods distribute color and contrast differently. No one of these no-reference criteria alone establishes color naturalness, artifact suppression, or recovery of the unknown scene radiance.

The prior-based results further clarify the cross-paradigm comparison. Among UDCP, RCP, and ULAP, UDCP leads in terms of UCIQE (0.524), UIQM (1.753), and URanker (2.052), RCP leads in terms of MUSIQ (42.002) and BC(e) (0.052), and ULAP leads in terms of BC(r) (7.750). DUIE exceeds these prior-based maxima by 1.456 in UIQM, 0.897 in URanker, 2.017 in MUSIQ, 0.090 in BC(e), and 10.897 in BC(r); only its UCIQE is 0.013 below UDCP. Taken together, the uniformly evaluated Sea-thru results show that DUIE offers the strongest overall rank profile in this comparison—coming first on four criteria, second on one, and third on one—while the criterion-specific exceptions remain important when interpreting perceptual quality.

### 5.6. Qualitative Comparisons

Figure 7 presents representative visual comparisons of all methods reported in Table 2 applied to three U45 scenes. The rows respectively contain a green-dominated stingray scene, a yellow–green seabed scene with shells and sea urchins, and a haze-dominated coral scene. Together, they expose differences in color correction, luminance allocation, local contrast, and texture retention under distinct degradations. Because no reference images are available for these scenes, the observations are descriptive and complement, rather than replace, the quantitative evaluation.

The learning-based baselines in Figure 7b–g exhibit different correction biases. U-shape substantially removes the dominant cast, but its second-row result is comparatively muted and some fine seabed variation is smoothed. PUGAN produces stronger local contrast and clearer object boundaries, yet introduces a warm yellow–brown tendency and drives several shell and coral highlights toward saturation. RLUIE is less consistent across the three cases: the first row retains a strong green cast, the second shifts toward yellow–green, and the third develops a pronounced pink–red bias that obscures the original tonal relationships. SMDR-IS, HCLR, and FUSION reveal more seabed and coral structure than the inputs, but their color and luminance distributions remain method-dependent. In particular, the shown HCLR result is relatively cool, whereas FUSION is warmer; both contain bright background regions and dark object areas whose contrast is stronger than in the input.

FAnNet, UDCP, RCP, ULAP, and Semi-UIR in Figure 7h–l exhibit a wide range of color and contrast restoration behavior. FAnNet retains considerable green content in the first two rows and shows visible chromatic speckling in the stingray scene, while the third-row coral region remains comparatively veiled. UDCP produces an aggressive contrast increase: it makes edges conspicuous, but the first row contains amplified colored grain and the second and third rows contain near-saturated highlights. RCP preserves more midtone structure, yet substantial cyan–green casts remain in all three examples. ULAP further increases separation between bright and dark regions, but the stingray and sea urchins become strongly compressed toward black and the first two scenes retain saturated green–cyan backgrounds. Semi-UIR achieves a more neutral overall balance and preserves recognizable object structure; however, its sandy and coral backgrounds are relatively bright, and some highlight detail is compressed. These examples illustrate why increased contrast or edge visibility alone cannot be equated with balanced color and natural luminance.

UIE-SAM and DUIE, shown in Figure 7m,n, provide the most complete cast correction among the displayed results, but their spatial allocation of enhancement differs. UIE-SAM creates high foreground–background separation: the first-row seabed becomes nearly white, several second-row shells are strongly brightened, and the third-row coral scene contains deep shadows adjacent to bright substrate. DUIE retains the corrected appearance while moderating these extremes in the shown cases. The first-row background preserves more tonal variation around the stingray, the second row retains clearer color differences among the shells, sand, and sea urchins, and the third row preserves coral texture with a less binary bright–dark distribution. Fine contours of the stingray and local structures in the shell-covered seabed and coral region remain visible. This behavior is consistent with the intended roles of RGB–depth continuous weighting, multi-scale candidate blending, and local refinement: the two region statistics candidates need not be selected as a hard final partition, and their contributions can vary gradually across space and scale.

These visual observations agree with the multi-criterion character of the U45 quantitative results: methods that create conspicuous contrast or saturated colors need not be preferred by the learned perceptual scorers, whereas DUIE is competitive across color, perceptual, and edge-based criteria. Nevertheless, Figure 7 contains only three representative scenes. It cannot independently attribute an observed difference to a single module, establish physical scene radiance recovery, or demonstrate superiority for every U45 image; controlled component-level evidence is therefore provided separately in Section 5.7.

### 5.7. Ablation Study

We evaluate the two trainable design components on Sea-thru by removing the pixel-level refinement module and the depth prior modeling mechanism individually and jointly. Table 5 reports the resulting four configurations. We further provide a qualitative ablation for three representative U45 images in Figure 8. The two experiments are complementary: the table measures average changes on Sea-thru, whereas the figure reveals spatial and appearance differences that are difficult to infer from scalar scores alone.

**(1)**  **Pixel-level refinement.** Removing pixel refinement while retaining depth modeling decreases URanker by 0.068, MUSIQ by 4.835, BC(e) by 0.009, and BC(r) by 1.062. The changes are consistent with the refinement network improving qualities captured by the learned and edge-based scorers. Because its residual is not constrained to a predefined frequency band, we interpret it as learned local correction rather than exclusively as high-frequency reconstruction.
**(2)**  **Depth prior modeling.** Removing depth prior modeling while retaining refinement reduces URanker by 0.011 and MUSIQ by 2.431; BC(e) is unchanged at the reported precision, and BC(r) decreases by 0.029. Thus, under this ablation, the main measured change appears in the learned quality scores rather than in edge count or gradient amplification. This pattern is consistent with the depth-conditioned coefficient affecting spatial blending, but it does not by itself prove the removal of every boundary artifact or physical correctness.
**(3)**  **Joint configuration.** Compared with the configuration that omits both modules, the complete model increases URanker by 0.085, MUSIQ by 5.344, BC(e) by 0.009, and BC(r) by 1.109. The complete configuration has the highest value in every reported column. These results support the complementarity of local learned refinement and continuous depth-conditioned fusion within the tested design; they do not imply that either depth guidance or NAF blocks are novel in isolation.
**(4)**  **Qualitative effects of refinement and fusion control.** All four enhanced variants substantially reduce the dominant green cast or haze relative to the inputs, confirming that the shared region statistics stage already supplies effective coarse color correction. Their differences are most evident in how that correction is distributed. Without both depth modeling and pixel refinement (Figure 8b), the correction is comparatively aggressive: the sand and shell highlights become strongly brightened, while the stingray, sea urchins, and several coral regions remain very dark. This larger bright–dark separation makes edges conspicuous, but it also compresses information in dark areas and produces a less even foreground–background balance. These visual tendencies are consistent with the lower MUSIQ and BC(r) values of the corresponding two-module removal in Table 5, although the different evaluation datasets preclude a sample-wise numerical correspondence.

Directly substituting the estimated relative depth map for the learned coefficient while retaining refinement (Figure 8c) recovers clear stingray contours, shell ridges, urchin spines, and coral structure. Nevertheless, visible cyan/green residuals remain in parts of the first two backgrounds, and the enhancement strength is not distributed as evenly as in the complete result. This comparison distinguishes merely possessing a depth cue from learning how RGB appearance and relative depth should jointly control candidate mixing: an uncalibrated monocular depth map encodes ordering, but its values are not themselves optimal enhancement coefficients.

The SAM mask composition with refinement (Figure 8d) also preserves substantially more local structure than the unrefined result, thereby isolating the contribution of pixel-level refinement from that of continuous fusion. However, a binary region assignment can select only one candidate at each location and cannot express gradual changes in enhancement strength within the same segmented region. In the displayed examples, this limitation appears as less uniform color and luminance transitions between salient objects and their surroundings, especially around the stingray and across the shell-covered seabed. The complete model (Figure 8e) instead combines both cues and presents the most spatially even balance among the shown variants while retaining the visible object contours and fine seabed structures. Together with Table 5, the figure supports a division of labor: refinement supplies local corrective capacity, whereas learned RGB–depth weighting and multi-scale fusion regulate where and how strongly the two interpretable candidates contribute. These observations are limited to the displayed cases and do not establish calibrated depth, exact physical restoration, or superiority for every scene.

### 5.8. Depth Estimation Robustness

To test sensitivity to errors in the internally estimated relative depth map, we perturb the depth input on U45 while leaving the RGB image, enhancement candidates, network parameters, and all other settings unchanged. The raw output $D_{0}$ of the frozen depth estimator is first normalized independently for each image:

(39) $$D\left(x\right)={\mathrm{clip}}_{\left[0,1\right]}\left(\frac{D_{0}\left(x\right)-D_{0,\min}}{D_{0,\max}-D_{0,\min}+10^{-6}}\right).$$

Thus, D(x)∈[0,1] preserves the relative ordering within an image but is not a metric depth measurement. Independent zero-mean Gaussian noise is then added at each pixel:

(40) $${\tilde{D}}_{\sigma }\left(x\right)={\mathrm{clip}}_{\left[0,1\right]}\left(D(x)+\epsilon (x)\right),\;\epsilon \left(x\right)∼N\left(0,\sigma_{D}^{2}\right),$$

The post-noise clipping retains the numerical range expected by the trained predictor; no second min–max normalization, smoothing, or denoising is applied to ${\tilde{D}}_{\sigma }$. The pseudo-random generator is initialized with seed 1234 for reproducibility. Because the clean map has a unit range, the nominal standard deviations $\sigma_{D}\in \left\{0.05,0.10,0.20\right\}$ represent 5%, 10%, and 20% of that range before clipping. The doubling schedule spans a mild perturbation, an intermediate level, and a deliberately strong unstructured perturbation while keeping the protocol compact. These values are sensitivity test levels rather than calibrated error estimates for a particular depth sensor or monocular estimator, see Table 6.

Here, ${\mathrm{MAE}}_{D}={\left|\Omega \right|}^{-1}\sum_{x\in \Omega }\left|{\tilde{D}}_{\sigma }\left(x\right)-D\left(x\right)\right|$, $\sigma_{\mathrm{eff}}$ is the empirical standard deviation of ${\tilde{D}}_{\sigma }-D$ after clipping and Clip is the fraction of perturbed pixels mapped to either boundary. These diagnostics confirm that the nominal and effective perturbations are not identical. When $\sigma_{D}$ increases from 0.05 to 0.20, the effective standard deviation increases from 0.0496 to 0.1758 rather than by the full nominal factor of four, while the boundary fraction rises from 0.9% to 15.4%. Hard clipping therefore progressively truncates the Gaussian tails and contributes to a sublinear increase in the perturbation delivered to the network.

The largest image quality transition nevertheless occurs between the structured unperturbed map and the first noisy condition. At $\sigma_{D}=0.05$, UCIQE and BC(e) change by +0.011 and +0.043, whereas URanker, MUSIQ, and BC(r) change by −0.085, −0.593, and −0.084, respectively. This clean-to-noisy transition is qualitatively different from increasing the amplitude of an already unstructured disturbance: independent noise disrupts the estimator’s spatially coherent relative depth pattern from the first nonzero level and changes the predicted mixing coefficient. The positive changes in UCIQE and BC(e) do not indicate more accurate depth or uniformly better restoration because those metrics reward particular changes in color contrast statistics or visible edges and need not decrease monotonically with depth error.

In contrast, increasing $\sigma_{D}$ from 0.05 to 0.20 produces little further variation in the reported image-domain means: the maximum difference is 0.001 for URanker and 0.005 for MUSIQ, while UCIQE, BC(e), and BC(r) are unchanged at three-decimal precision. The depth-domain diagnostics show that this plateau is not caused by identical perturbed maps; the input disturbance continues to grow. Rather, the observation is consistent with several attenuation mechanisms in the inference path. The depth map is interpreted jointly with unchanged RGB evidence instead of being used directly as the restored image. Spatial convolutions can reduce the influence of independent zero-mean fluctuations, the post-noise clip and the final Sigmoid keep the input and predicted coefficient bounded, and the Gaussian pyramid low-pass filters the coefficient at progressively coarser fusion scales. Moreover, the coefficient only adjusts the proportions of two fixed enhanced candidates; it does not generate unconstrained color or texture. The fixed high-frequency fusion stage is also identical for every $\sigma_{D}$ and reintroduces the same RGB-derived structural signal. These mechanisms provide plausible explanations for why a growing pixel-level depth disturbance produces a much smaller change in aggregate image quality scores, although the present experiment does not isolate their individual causal contributions.

Dataset averaging over 45 images and reporting to three decimals further conceal small image-specific variations. Thus, the near-equal rows should be interpreted as an output-level plateau under this particular protocol, not as evidence that $\sigma_{D}=0.05$, 0.10, and 0.20 produce equivalent depth maps. At $\sigma_{D}=0.20$, the changes relative to the unperturbed output remain +0.011 for UCIQE, −0.084 for URanker, −0.597 for MUSIQ, +0.043 for BC(e), and −0.084 for BC(r). The experiment supports limited sensitivity to independent, zero-mean Gaussian perturbations after per-image normalization and hard clipping. It does not establish robustness to biased or spatially correlated errors, large-scale reversal of near–far ordering, missing structures, or domain shifts in the frozen estimator, which could alter the fusion coefficient coherently and need not be attenuated in the same way.

## 6. Discussion

The experimental results provide evidence for the effectiveness of DUIE, but their principal value is to clarify how its components cooperate. As summarized in Table 1, the framework is best understood as a complementary tri-paradigm integration of physics-inspired, statistical, and learned components. The three paradigms do not operate as interchangeable enhancement branches. Instead, they contribute different forms of prior knowledge and are connected through an explicit coarse-to-fine processing path: underwater image formation supplies the spatial degradation motivation, regional statistics construct interpretable enhancement candidates, and learned modules refine and continuously combine those candidates. This organization places DUIE between a fully hand-crafted inverse model and an unconstrained end-to-end mapping.

**Limitations of Isolated Paradigms.** Physical model-based methods relate the observed image to scene radiance through transmission, background light, attenuation, and propagation distance [10,13]. Their parameters are physically meaningful, but practical restoration can be sensitive to model assumptions, water type, illumination, depth availability, and estimation error. In complex or spatially heterogeneous scenes, inaccurate parameters may propagate through the inverse process and leave residual color casts, haze, or amplified artifacts. Moreover, methods that require RGB-D measurements are not directly applicable when only a single RGB image is available.

Non-physical statistical methods avoid explicit inversion and consequently retain useful flexibility and training-free robustness. Fusion, histogram, Retinex, and region statistics approaches can correct conspicuous color imbalance without estimating every optical parameter [16,31,32]. However, image-level operations apply one correction tendency to the entire scene, while a small number of discrete regions provides only coarse spatial control. Neither representation naturally describes a gradual near–far change in degradation strength. When the foreground and background require different correction magnitudes, global processing may over-enhance nearby content or under-enhance distant content, whereas hard region assignment can introduce abrupt transitions near mask boundaries.

End-to-end deep models offer strong nonlinear representation capacity and can learn complex local transformations from data [40,43]. Nevertheless, their behavior depends on the supervision and training distribution, and the relation between an internal feature response and underwater image formation is often difficult to interpret. A model may also optimize the attributes favored by its objective or evaluation distribution without preserving the desired balance among color, contrast, structure, and natural appearance. The differing UCIQE, UIQM, URanker, and MUSIQ rankings in Table 2, Table 3 and Table 4 illustrate this broader point: no individual statistical or learned score fully characterizes enhancement quality.

**Complementary Tri-Paradigm Integration.** DUIE is designed to alleviate these isolated limitations by assigning a targeted role to each paradigm rather than asking one paradigm to solve the complete restoration problem. The integration can be interpreted through three complementary properties:

**(1)**  **Adherence to depth-dependent optical degradation (physics-inspired integration).** The underwater formation model indicates that attenuation changes with propagation distance and wavelength. DUIE converts this insight into an architectural prior: a frozen monocular estimator supplies relative near–far ordering, and the trainable predictor combines that ordering with RGB appearance to generate a continuous mixing coefficient. The coefficient controls how strongly the two enhancement candidates contribute at each location and across Laplacian pyramid scales. This design is more closely aligned with spatially varying degradation than a global coefficient or binary final mask. However, the formation model is not explicitly inverted, and the predictor does not estimate calibrated attenuation coefficients, transmission, absolute metric depth, or scene radiance. Thus, “physics-inspired” describes the source of the spatial prior rather than a claim of physically exact recovery.
**(2)**  **Preservation of practical generality (statistical integration).** Foreground- and background-adaptive color compensation produces two explicit candidates with different correction tendencies. Because these candidates are constructed from region statistics instead of regenerated from an unconstrained latent representation, their roles remain inspectable and can be traced to the inherited color compensation procedure. The statistical stage supplies a stable macroscopic starting point, while continuous learned mixing prevents either candidate from being imposed uniformly. In this sense, DUIE retains the operational simplicity of non-physical enhancement while using relative depth to overcome its coarse spatial control.
**(3)**  **Fine-grained texture reconstruction (learned integration).** The NAF-based refinement networks learn residual corrections for the two statistically enhanced candidates. Their purpose is not to reconstruct the entire color distribution from scratch, but to recover local detail and correct artifacts that fixed analytical operations do not adequately describe. The subsequent Laplacian pyramid fusion propagates the learned coefficient from coarse appearance to fine spatial scales, thereby connecting macroscopic color adjustment with local texture preservation. It also softens the transition between candidates compared with direct binary composition.

The ablation results support this division of labor. Removing pixel refinement causes the largest measured reductions in MUSIQ and BC(r), which is consistent with the refiner improving attributes captured by learned quality and edge visibility assessment. Removing depth conditioning primarily changes the learned quality scores while leaving BCRA comparatively stable at the reported precision, suggesting that its principal role is spatial selection rather than indiscriminate edge amplification. The configuration containing both components produces the strongest joint result in Table 5; therefore, the evidence favors their complementarity rather than treating either component as a complete enhancement solution by itself.

The cross-dataset results provide a second perspective on this complementarity. DUIE obtains the highest UIQM on U45, SUIM, and Sea-thru and leads in terms of MUSIQ and both edge visibility descriptors for U45 and SUIM. On Sea-thru, it ranks first in URanker and both BCRA descriptors, second in MUSIQ, and third in UCIQE; UDCP leads in UCIQE, whereas HCLR leads in MUSIQ. UCIQE and UIQM aggregate different color contrast attributes, BCRA responds to visible-edge changes, and URanker and MUSIQ embody different learned preferences. The absence of a single universal ordering is consequently expected. DUIE’s contribution is better characterized by its explicit allocation of functions and its consistently strong multi-criterion profile than by a claim of superiority under every scalar metric.

Overall, DUIE constitutes a grey box enhancement framework: it preserves the interpretability of a depth-dependent degradation motivation and explicit regional candidates, retains the practical robustness of statistical preprocessing, and introduces learned nonlinear capacity where fixed operations are least expressive. The resulting tri-paradigm organization offers a coherent route from coarse region-aware correction to fine, continuously controlled reconstruction. Its conclusions remain confined to perceptual underwater image enhancement; they do not imply calibrated physical inversion or verified improvement in downstream perception systems.

## 7. Conclusions and Future Work

This study is motivated by the depth-dependent nature of underwater optical degradation. Existing enhancement methods driven mainly by global or region-level statistics often lack explicit depth-aware guidance, resulting in a mismatch between enhancement intensity and spatially varying degradation and thus compromising the spatial coherence and visual naturalness of the restored images. To address these limitations, we propose a novel Depth-Aware Underwater Image Enhancement (DUIE) framework. Specifically, we first introduce a depth prior modeling mechanism that dynamically translates relative geometric depth into a spatially continuous fusion weight map. Then, we propose an NAF-powered pixel-level refinement module to meticulously reconstruct high-frequency structural details. Ultimately, we design a depth-weighted Laplacian pyramid fusion strategy to seamlessly synthesize the refined foreground and background enhanced images. Comprehensive qualitative and quantitative experiments across multiple challenging datasets validate the superiority of our proposed method in rectifying severe color deviations and restoring local textures. By shifting the enhancement paradigm from coarse-grained region-level control to fine-grained, depth-guided spatially adaptive enhancement, our method produces more physically coherent visual results and reduces boundary artifacts. It establishes a highly physically plausible and structurally robust visual preprocessing foundation, offering a mission-critical tool for downstream applications including Autonomous Underwater Vehicle (AUV) navigation, subsea inspection, and marine ecological observation.

Although our method performs exceptionally well in addressing complex underwater degradation, there is still room for further improvement. First, DUIE depends on the domain generalization of two frozen components. Severe turbidity, low texture, unusual lighting, or an incorrect near–far ordering from WsUID-Net can produce suboptimal fusion weights; weak foreground–background separation from the SAM can also degrade the two regional candidates. The Gaussian perturbation test in Section 5.8 covers independent noise on normalized depth but does not resolve structured depth errors, incorrect ordering, or domain shift. Future evaluation will therefore include structured perturbations and representative failure cases, together with alternatives for confidence-aware depth conditioning and more reliable region cues. Second, UIEB reference images are human-selected perceptual targets rather than measurements of physical scene radiance, and the pixel-wise MSE favors average fidelity without directly optimizing perceptual naturalness or downstream utility. Complementary perceptual or structure-aware objectives may improve this balance, but their effect must be established through controlled ablation. Paired PSNR/SSIM evaluation and downstream task assessment should likewise be reported only when DUIE and every comparator are evaluated on the same split and protocol. Third, the frozen SAM and depth networks, two candidate refinement paths, and multi-scale pyramid processing introduce computation and memory overhead. Because the present study does not report run-time, energy consumption, or deployment measurements, no real-time claim is made. Future work will measure these costs on common hardware and investigate parameter sharing, lightweight backbones, model compression, and knowledge distillation [69,70,71] before drawing conclusions about resource-constrained deployment. Finally, the color compensation thresholds, pyramid level, and edge fusion parameters are empirically fixed. Their sensitivity and transferability across water types remain to be quantified. Future work will examine systematic hyperparameter sensitivity, adaptive parameter prediction, broader cross-domain evaluation, and joint optimization of the statistical and learned stages. These directions aim to improve adaptability while preserving the explicit division of labor that defines the tri-paradigm framework.

## Figures and Tables

**Figure 1:**
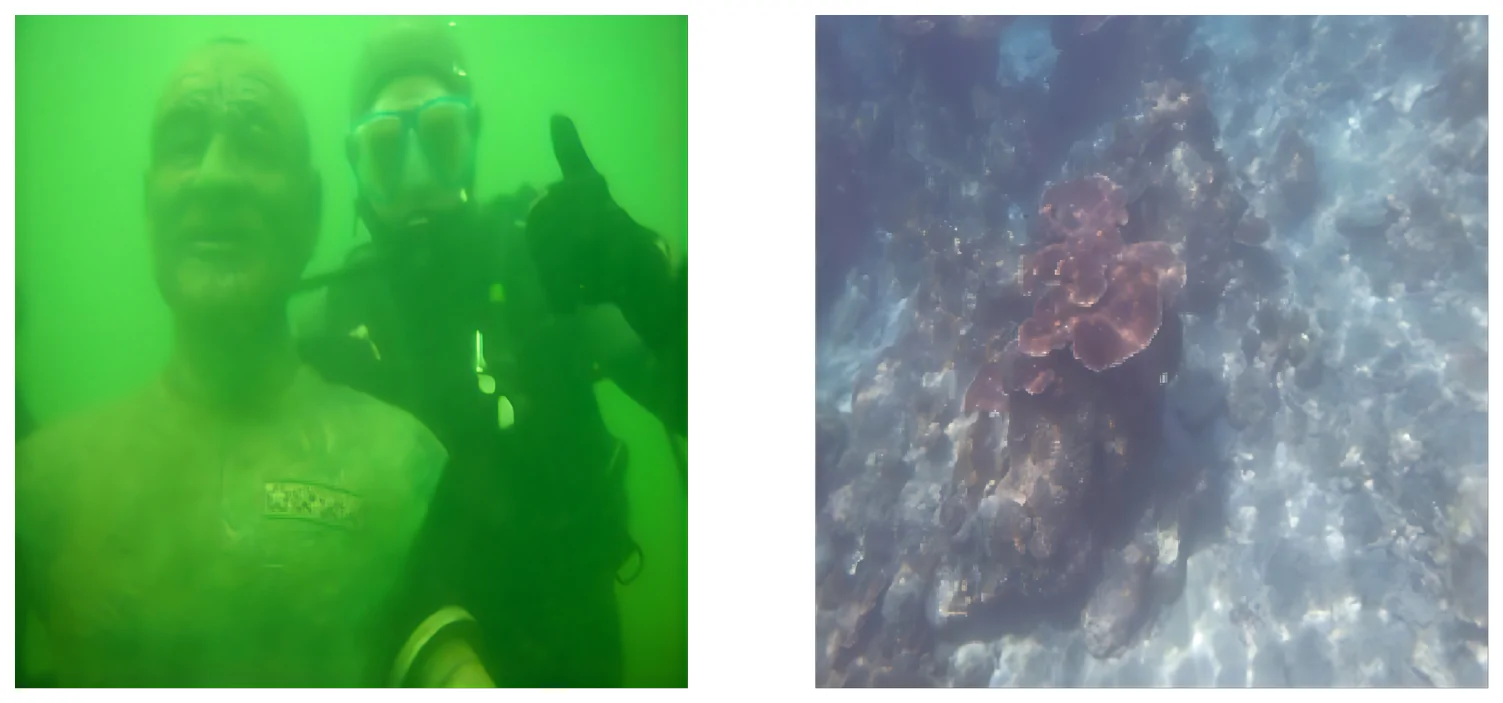
Typical visual degradations in underwater environments. (**Left**) Green color cast caused by wavelength-dependent light absorption. (**Right**) Haze, low contrast, and structural blur caused by light scattering and suspended particles.

**Figure 2:**
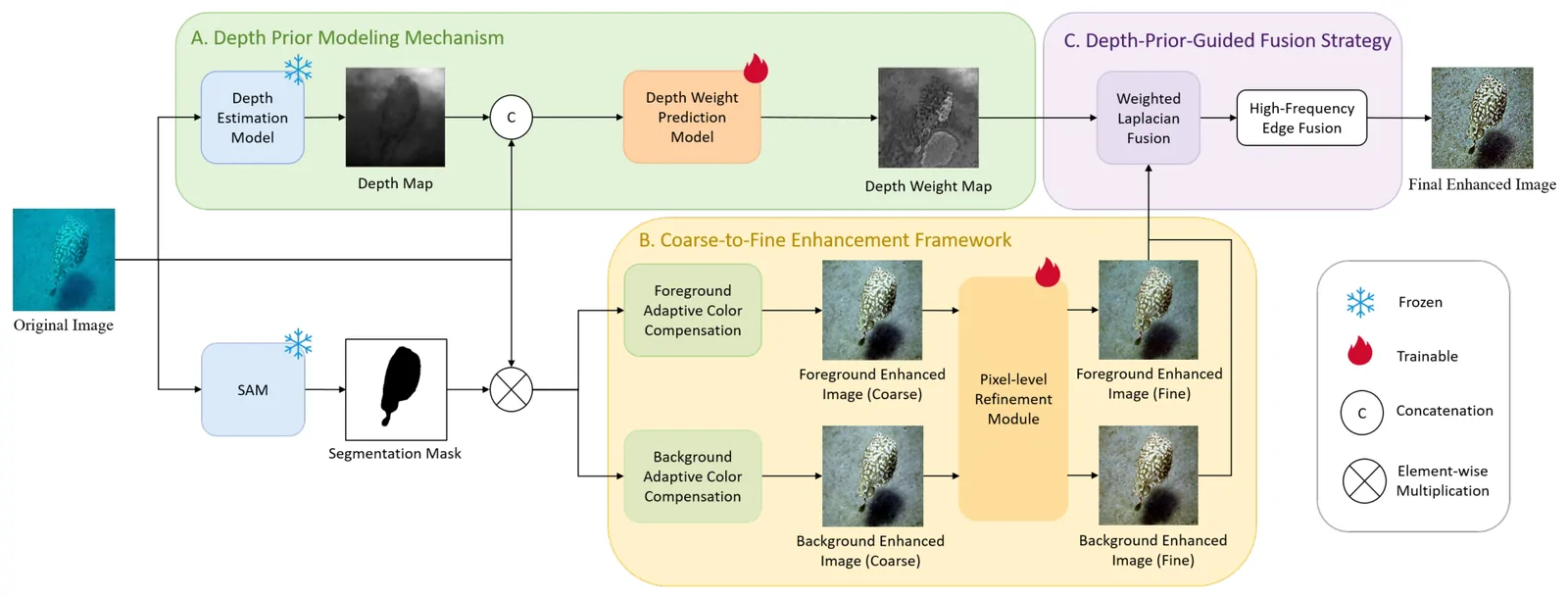
Overall DUIE architecture. A frozen depth estimator and frozen SAM derive geometric and region cues from a single RGB input. The trainable RGB–depth predictor generates a continuous fusion coefficient, while the trainable pixel refiner processes two statistically compensated candidates before multi-scale fusion.

**Figure 3:**
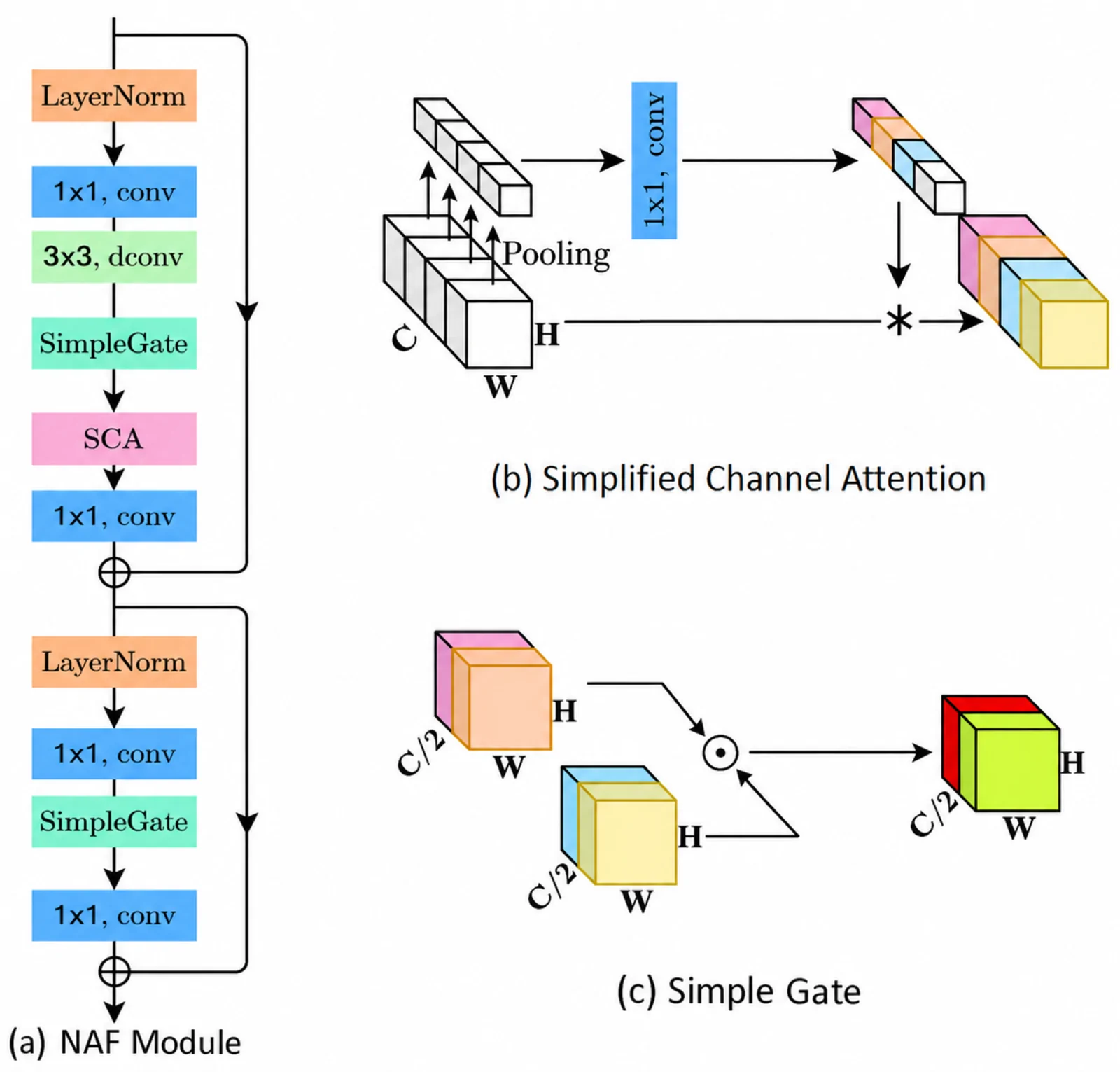
The architecture of the Nonlinear Activation-Free (NAF) module [46]. (**a**) The overall structure of the NAF block, which discards conventional activation functions. (**b**) The Simplified Channel Attention (SCA) module for efficient global context aggregation. (**c**) The SimpleGate mechanism, which performs channel-wise splitting and element-wise multiplication.

**Figure 4:**
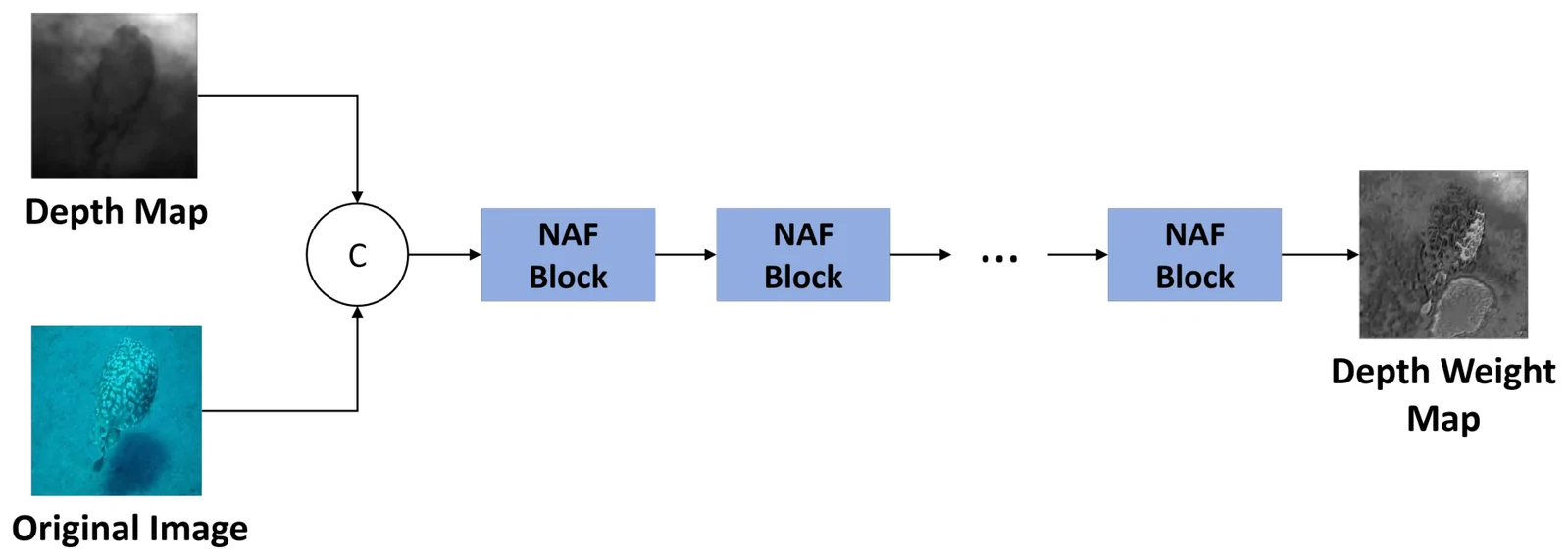
Network architecture of the depth weight prediction module. The module concatenates the input image and estimated relative depth, then uses lightweight NAF blocks to predict a continuous candidate fusion coefficient.

**Figure 5:**
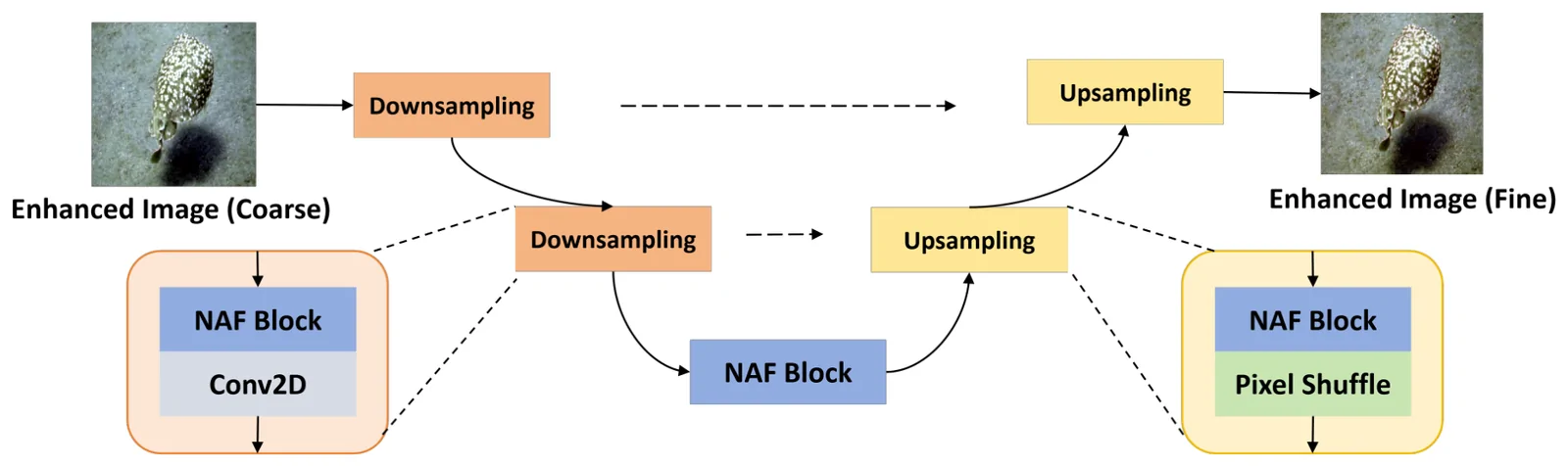
Network architecture of the pixel-level refinement module. It uses a U-Net-style encoder–decoder built from NAF blocks, PixelShuffle upsampling, and a global residual connection to learn local candidate corrections.

**Figure 6:**
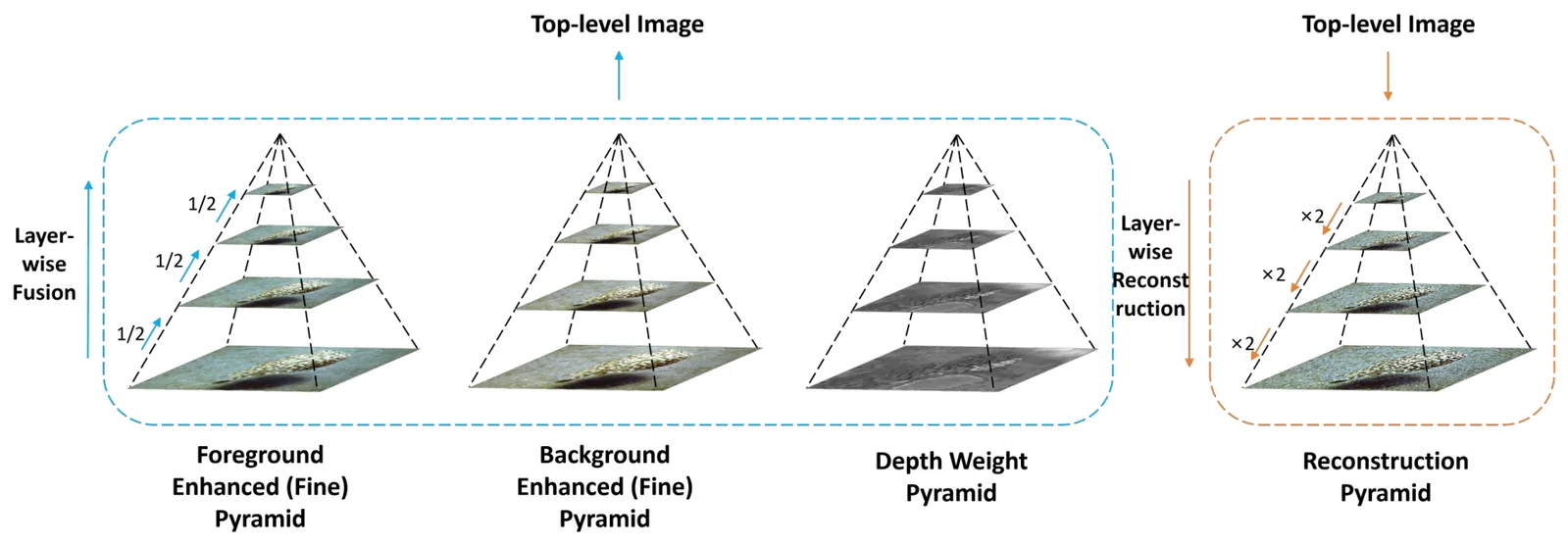
Depth-weighted Laplacian pyramid fusion. Foreground and background candidate Laplacian levels are blended using the corresponding Gaussian pyramid level of the continuous coefficient and then reconstructed from coarse to fine.

**Figure 7:**
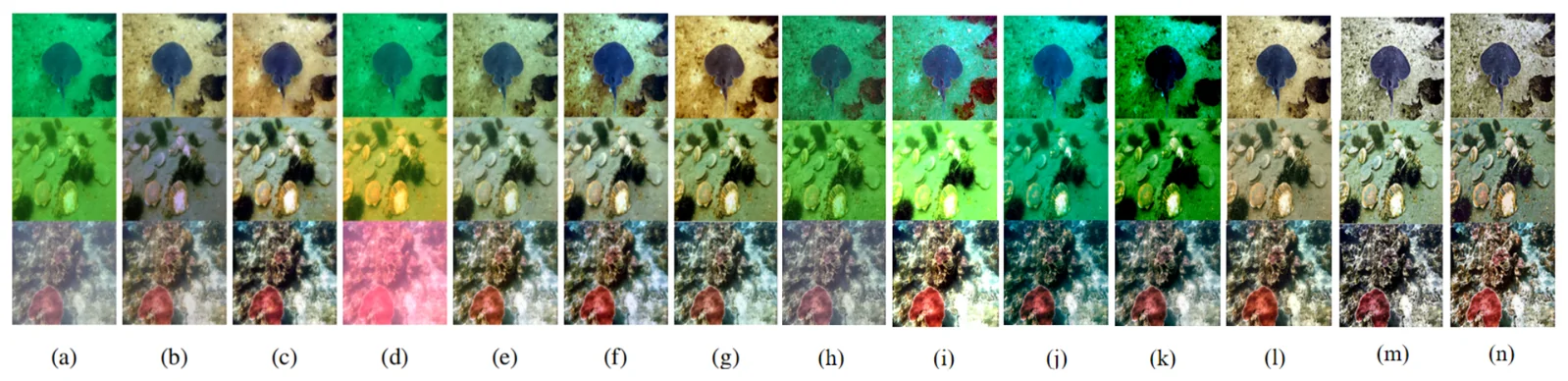
Qualitative comparison on U45. (**a**) Input. (**b**) U-shape [43]. (**c**) PUGAN [40]. (**d**) RLUIE [59]. (**e**) SMDR-IS [60]. (**f**) HCLR [44]. (**g**) FUSION [61]. (**h**) FAnNet [39]. (**i**) UDCP [20]. (**j**) RCP [21]. (**k**) ULAP [22]. (**l**) Semi-UIR [62]. (**m**) UIE-SAM [16]. (**n**) DUIE. The rows show green-, yellow–green-, and haze-dominated examples, respectively.

**Figure 8:**
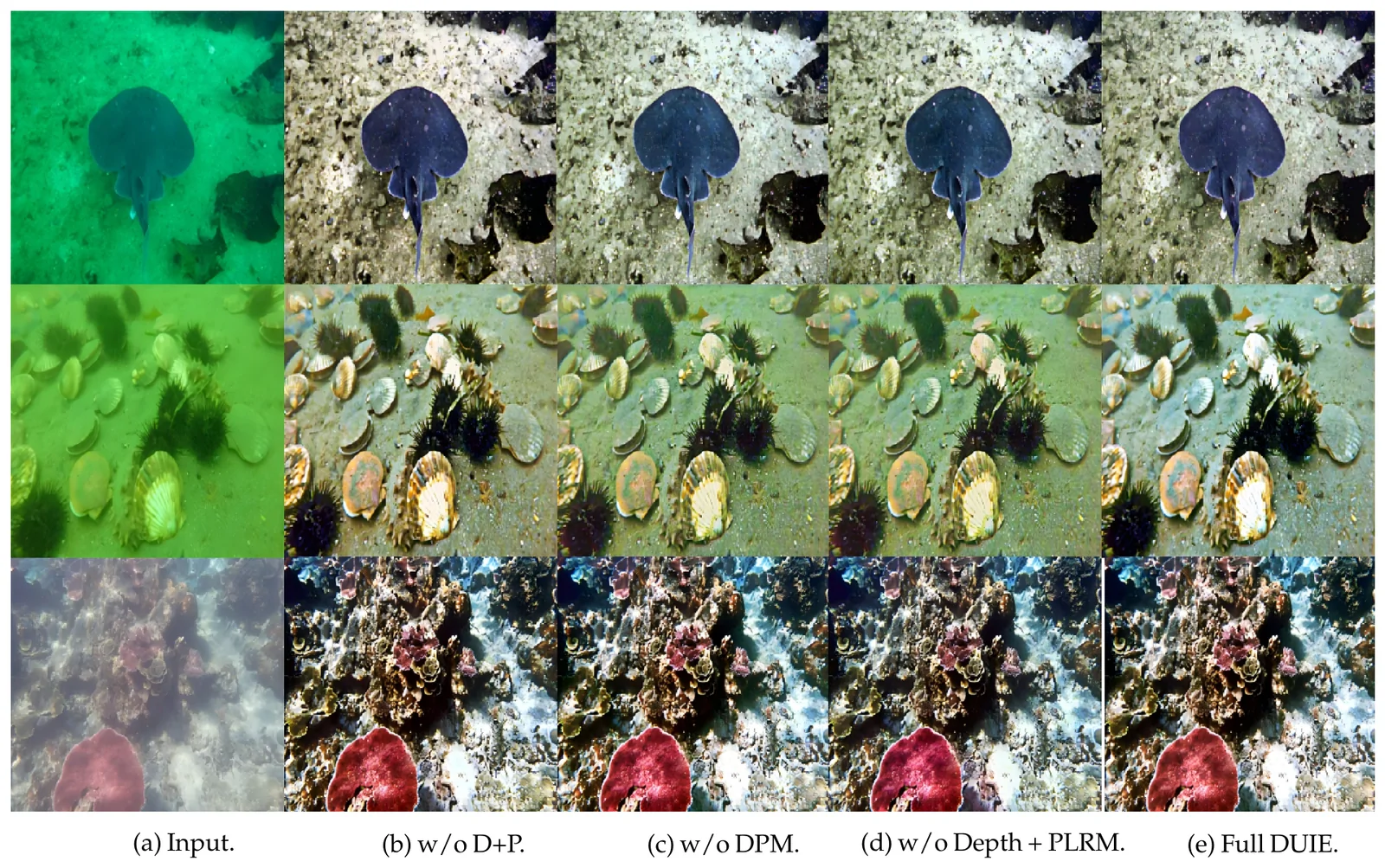
Qualitative ablation comparison for three representative U45 scenes. From top to bottom, the rows show two green/yellow–green color cast scenes and one low-contrast hazy scene. (**a**) Raw input. (**b**) w/o D + P: both depth prior modeling and pixel-level refinement are removed, and the two coarse candidates are composed with the SAM mask. (**c**) w/o DPM: pixel-level refinement is retained, but the raw estimated relative depth map is directly used as the mixing weight instead of the learned RGB–depth predictor. (**d**) w/o Depth + PLRM: pixel-level refinement is retained, but the binary SAM mask is used for candidate composition. (**e**) Complete DUIE. DPM and PLRM denote depth prior modeling and pixel-level refinement, respectively.

**Table 1:** Schematic comparison of representative underwater image enhancement paradigms. “Derived cues” are estimated internally from the same RGB image and are not independent sensing modalities.

Category	Representative Methods	Input and Output Model	Main Advantages	Main Limitations	Relation to DUIE
Physical model-based	UDCP, ULAP, Sea-thru [13,20,22]	RGB or RGB-D → estimated optical parameters and scene radiance	Explicit relation to image formation; interpretable parameters	Sensitive to assumptions and parameter errors; RGB-D may require another sensor	Supplies the depth-dependent attenuation motivation; DUIE does not invert this model
Non-physical model-based	Retinex, fusion, UIE-SAM [16,31,32]	RGB → transformed or fused RGB	Transparent image operations; no full optical inversion; little or no paired training	Image-level or discrete region statistics may not track gradual depth variation	UIE-SAM supplies the two region-statistics candidates; DUIE replaces its binary final mixing control
Deep learning-based	WaterNet, PUGAN, SARSDN, Depth-Conditioned GAN, UVZ [17,18,19,36,40]	RGB, or RGB with derived priors → enhanced RGB	Flexible nonlinear local and global feature modeling	Depends on supervision and training distribution; internal decisions can be difficult to interpret	Establishes learned enhancement and depth conditioning; DUIE assigns learning to refinement and explicit candidate mixing
DUIE	Proposed framework	Single RGB; internally derived SAM mask and relative depth → enhanced RGB	Interpretable candidates with learned continuous, multi-scale fusion control	Depends on frozen SAM/depth priors and empirical parameters; no full physical inversion	Combines the three families while keeping their roles explicit

**Table 2:** Quantitative comparison on U45. Bold and underlined values denote the best and second-best results, respectively, among methods evaluated on all 45 images.

Methods	UCIQE ↑	UIQM ↑	URanker ↑	MUSIQ ↑	BC(e) ↑	BC(r) ↑
U-shape [43]	0.370	3.190	1.242	43.464	0.496	1.687
PUGAN [40]	0.427	3.259	2.069	44.890	0.764	2.068
RLUIE [59]	0.265	2.476	−1.133	38.054	−0.237	1.002
SMDR-IS [60]	0.380	3.212	1.741	48.512	0.705	1.939
HCLR [44]	0.425	3.245	1.997	48.036	0.765	2.066
FUSION [61]	0.401	3.081	1.652	50.382	0.747	1.975
FAnNet [39]	0.301	2.722	0.487	49.741	0.703	1.785
UDCP [20]	0.414	2.313	1.001	45.463	0.756	2.266
RCP [21]	0.337	1.617	0.500	46.264	0.715	1.534
ULAP [22]	0.432	1.583	0.838	47.068	0.889	2.021
UIE-SAM [16]	**0.463**	2.680	**2.797**	52.378	1.383	4.671
Semi-UIR [62]	0.402	3.251	2.165	49.252	0.882	2.352
**Ours**	0.462	**3.331**	2.611	**53.547**	**1.471**	**4.725**

**Table 3:** Quantitative comparison on SUIM. Bold and underlined values denote the best and second-best available results, respectively.

Methods	UCIQE ↑	UIQM ↑	URanker ↑	MUSIQ ↑	BC(e) ↑	BC(r) ↑
U-shape [43]	0.401	2.585	1.181	43.496	0.005	1.345
PUGAN [40]	0.457	2.550	1.719	42.543	0.200	1.530
RLUIE [59]	**0.532**	2.684	1.063	37.091	0.484	2.251
SMDR-IS [60]	0.425	2.542	1.433	59.747	0.352	1.518
HCLR [44]	0.443	2.565	1.579	44.956	0.105	1.516
FUSION [61]	0.432	2.587	1.356	45.934	−0.038	1.152
FAnNet [39]	0.383	2.378	1.167	61.231	0.522	1.665
UDCP [20]	0.458	2.156	1.187	57.333	0.599	1.869
RCP [21]	0.338	1.420	0.486	55.599	0.366	1.124
ULAP [22]	0.474	0.976	0.726	55.162	0.651	1.147
UIE-SAM [16]	0.482	2.219	**2.182**	60.912	1.304	3.735
Semi-UIR [62]	0.445	2.619	1.659	61.175	0.528	1.835
**Ours**	0.477	**2.782**	2.065	**61.963**	**1.563**	**3.918**

**Table 4:** Quantitative comparison on Sea-thru. All methods are evaluated on 1216 images. Bold and underlined values denote the best and second-best results, respectively.

Methods	UCIQE ↑	UIQM ↑	URanker ↑	MUSIQ ↑	BC(e) ↑	BC(r) ↑
U-shape [43]	0.337	3.144	0.871	25.015	−0.109	2.297
PUGAN [40]	0.488	2.609	−0.477	19.319	−0.896	2.754
RLUIE [59]	0.273	2.743	−1.495	20.297	−0.613	0.426
SMDR-IS [60]	0.205	2.433	0.280	38.331	0.049	2.712
HCLR [44]	0.348	3.011	1.381	**47.259**	−0.087	4.637
FUSION [61]	0.246	2.646	−0.418	27.664	−0.946	2.217
FAnNet [39]	0.102	1.613	−0.792	33.857	0.069	1.447
UDCP [20]	**0.524**	1.753	2.052	38.096	0.042	7.401
RCP [21]	0.320	1.146	0.308	42.002	0.052	4.793
ULAP [22]	0.434	0.260	0.532	37.172	−0.598	7.750
UIE-SAM [16]	0.521	2.446	2.864	38.674	0.125	17.586
Semi-UIR [62]	0.407	3.000	1.882	41.416	0.074	6.809
**Ours**	0.510	**3.209**	**2.949**	44.019	**0.141**	**18.647**

**Table 5:** Ablation study results on the Sea-thru dataset. The best results are highlighted in bold.

Pixel-Level Refinement	Depth Prior Modeling	URanker ↑	MUSIQ ↑	BC(e) ↑	BC(r) ↑
✓	✓	**2.949**	**44.018**	**0.141**	**18.647**
×	✓	2.881	39.183	0.132	17.585
✓	×	2.938	41.587	0.141	18.618
×	×	2.864	38.674	0.132	17.538

**Table 6:** Sensitivity to Gaussian perturbations of the normalized relative depth map on U45. Depth-domain diagnostics are computed after the post-noise clipping. The unperturbed row is the reported DUIE result in Table 2.

$\sigma_{D}$	${\mathrm{MAE}}_{D}$	$\sigma_{\mathrm{eff}}$	Clip (%)	UCIQE ↑	URanker ↑	MUSIQ ↑	BC(e) ↑	BC(r) ↑
0 (unperturbed)	0.0000	0.0000	0.0	0.462	2.611	53.547	1.471	4.725
0.05	0.0396	0.0496	0.9	0.473	2.526	52.954	1.514	4.641
0.10	0.0774	0.0962	4.9	0.473	2.526	52.949	1.514	4.641
0.20	0.1419	0.1758	15.4	0.473	2.527	52.950	1.514	4.641

## Data Availability

The public datasets analyzed in this study are available from their original sources cited in the manuscript. Additional implementation details and generated data are available from the corresponding author upon reasonable request.

## Abbreviations

The following abbreviations are used in this manuscript:

AUV	Autonomous Underwater Vehicle
BC(e)	Increase in visible edges
BC(r)	Change in edge pixel gradients
BCRA	Blind Contrast Restoration Assessment
DUIE	Depth-Aware Underwater Image Enhancement
MSE	Mean Squared Error
MUSIQ	Multi-Scale Image Quality Transformer
NAF	Nonlinear Activation Free
SAM	Segment Anything Model
SLAM	Simultaneous Localization and Mapping
UCIQE	Underwater Color Image Quality Evaluation
UIE	Underwater Image Enhancement
UIQM	Underwater Image Quality Measure
URanker	Ranking-Based Underwater Image Quality Assessment
